# Long-Term Metabolic Responses of Olive to Bacterial and Fungal Inoculation Differ Between Cultivars

**DOI:** 10.3390/biom16081220

**Published:** 2026-08-21

**Authors:** Sergeja Adamič Zamljen, Sara Godena, Nikola Major, Smiljana Goreta Ban, Tvrtko Karlo Kovačević, Marija Polić Pasković, Igor Pasković

**Affiliations:** 1 Department of Agronomy, Biotechnical Faculty, University of Ljubljana, Jamnikarjeva 101, 1000 Ljubljana, Slovenia; sergeja.adamiczamljen@bf.uni-lj.si; 2 Department of Agriculture and Nutrition, Institute of Agriculture and Tourism, Karla Huguesa 8, 52440 Poreč, Croatia; nikola@iptpo.hr (N.M.); smilja@iptpo.hr (S.G.B.); tvrtko@iptpo.hr (T.K.K.); paskovic@iptpo.hr (I.P.)

**Keywords:** *Olea europaea* L., olive leaves, plant–pathogen interaction, primary metabolites, phenolic compounds, oxidative stress, lipid peroxidation, metabolite profiling

## Abstract

Olive leaves represent a metabolically active tissue that plays an important role in plant responses to biotic stress. The present study comprised two independent experiments investigating biochemical responses of olive leaves to bacterial and fungal challenge under controlled conditions. Changes in primary metabolites (sugars, organic acids and free amino acids), phenolic compounds and lipid peroxidation were analyzed using chromatographic and spectrophotometric methods. In the bacterial experiment, pronounced differences were observed in primary metabolism. Tryptophan concentrations ranged from approximately 50 mg kg^−1^ DW to more than 360 mg kg^−1^ DW in ‘Istarska bjelica’, while sucrose concentrations reached up to 87 g kg^−1^ DW, demonstrating cultivar-dependent differences in carbohydrate metabolism. Phenolic profiling showed that secoiridoids were the dominant phenolic class, with oleuropein concentrations exceeding 27 g kg^−1^ DW across bacterial treatments. In the fungal experiment, amino acids showed greater variability than sugars and phenolic compounds, whereas MDA concentrations ranged from approximately 190 to 300 nmol g^−1^ DW but did not differ significantly among pathogen treatments. Overall, the two experiments showed distinct patterns of metabolite variation associated with bacterial and fungal challenge. These findings contribute to a better understanding of cultivar-dependent metabolic responses and provide a basis for future studies of olive–microbe interactions.

## 1. Introduction

The olive tree (*Olea europaea* L.) is a perennial crop of significant importance in the Mediterranean region. It plays a central role in agricultural production, landscape management, and cultural heritage. In addition to olive oil and table olives, various parts of the olive tree have attracted increasing scientific attention due to their rich chemical composition and biological activity. Of these tissues, olive leaves represent a significant source of bioactive compounds, including phenolics, sugars, organic acids and amino acids, which contribute to the physiological functioning of the plant and may play a role in stress responses [1,2].

Plant–microbe interactions frequently induce substantial metabolic reprogramming in plant tissues. In response to pathogen attack, plants initiate complex defensive mechanisms involving the coordinated regulation of both primary and secondary metabolism. Primary metabolites, including sugars, organic acids and amino acids, have been shown to provide essential metabolic energy and biosynthetic precursors required for the synthesis of defense-related compounds [3,4]. In addition to this primary function, these metabolites also act as signaling molecules that regulate plant responses to stress. Numerous metabolomic studies have demonstrated that pathogen infection can strongly influence plant metabolic profiles, resulting in alterations in carbohydrate metabolism, amino acid pools and other primary metabolites that are associated with plant defense responses [4,5,6].

In olive trees, metabolomic investigations have revealed that primary metabolites constitute a significant proportion of the leaf metabolome, with this proportion potentially varying depending on environmental conditions, the genotype and the physiological status of the plant [2,7,8]. Sugars such as sucrose and glucose have been demonstrated to play an important role in carbon allocation and metabolic regulation during stress conditions [5,6]. In contrast, amino acids such as glutamate and tryptophan have been shown to be key intermediates linking primary metabolism with the biosynthesis of specialized metabolites involved in plant defense. Despite their importance, relatively few studies have investigated how primary metabolite profiles in olive leaves respond to pathogen infection [2,8]. In addition to primary metabolites, a high concentration of phenolic compounds is observed in olive leaves, constituting one of the most significant groups of secondary metabolites present in olive tissues. These compounds include secoiridoids, flavonoids and phenolic acids, many of which possess strong antioxidant and antimicrobial properties [1,8,9]. Among them, oleuropein is recognized as the dominant phenolic compound in olive leaves and is widely considered a key component of the biochemical defense system of olive trees [2,10]. Variations in oleuropein and related secoiridoids have been associated with environmental stress, pathogen infection and genetic differences among cultivars [11,12,13]. Another significant aspect of plant responses to microbial infection is oxidative stress. In the course of plant–pathogen interactions, the production of reactive oxygen species (ROS) frequently constitutes an integral component of the initial defense response. While ROS can contribute to pathogen restriction, excessive accumulation may lead to oxidative damage of cellular components, including membrane lipids [14,15]. The evaluation of lipid peroxidation is typically achieved through the accumulation of malondialdehyde (MDA), which functions as a widely accepted indicator of oxidative stress in plant tissues [16].

Despite the fact that a number of studies have investigated the phenolic composition and antioxidant properties of olive leaves, relatively little is known about the integrated metabolic responses of olive leaves to microbial challenges involving both primary metabolites, secondary metabolites and oxidative stress indicators [2,8]. In particular, the metabolic responses of olive leaves to different types of microbial agents, including bacterial isolates and fungal pathogens, remain insufficiently explored [3,4]. It is also noteworthy that the metabolic response of olive leaves may be subject to variation depending on cultivar. It is evident that olive cultivars frequently demonstrate considerable variations in physiological traits, metabolic composition and stress tolerance. As demonstrated in previous studies, there is considerable potential for variation in the phenolic profiles and other metabolic characteristics of olive leaves among different cultivars, indicative of genetic variability within the species [12,13]. In the present study, three traditional Istrian cultivars—‘Istarska bjelica’, ‘Rosinjola’ and ‘Buža’—that are widely cultivated in the northern Adriatic region were selected for analysis because they represent economically important regional olive varieties, which are known to differ in biochemical composition and physiological characteristics. These cultivars were also selected based on their response to various biotic stresses in previous research related to bacterial and fungal olive pathogens [17,18].

The present study comprised two independent experimental systems designed to investigate metabolic responses of olive leaves to microbial challenge. In the first experiment, olive leaves were exposed to two isolates of *Pseudomonas savastanoi* pv. *savastanoi* (*Pss*), whereas the second experiment evaluated responses to two fungal pathogens, *Neofusicoccum parvum* and *Dothiorella iberica*. Primary metabolites, phenolic compounds and lipid peroxidation were analyzed to characterize metabolic responses within each experimental system. In addition, cultivar-dependent differences in metabolite profiles were evaluated using univariate and multivariate analyses. The aim of this study was to characterize metabolic responses of olive leaves to bacterial and fungal challenge in two independent experimental systems and to identify metabolites associated with cultivar-dependent responses.

## 2. Materials and Methods

### 2.1. Experimental Design

Olive seedlings from two independent greenhouse pathogenicity experiments previously established at the Institute of Agriculture and Tourism were used for the present metabolomic analyses. The greenhouse experiment consisted of olive seedlings from 2–3 years old (*Pss*) to five years old (fungi). The average temperature during the experimental period in the greenhouse ranged between 24 and 25 °C, with a relative humidity of 85%. Plants were irrigated using a drip irrigation system, ensuring that all plants received the same amount of water, while photoperiod followed natural distribution.

In the bacterial experiment, ‘Istarska bjelica’ and ‘Rosinjola’, previously identified as the least and most susceptible cultivars, respectively, were inoculated with *Pseudomonas savastanoi* pv. *savastanoi* isolates B45 and I7, representing the least (112.39 ± 7.29) and most virulent isolates (258.39 ± 9.00) with respect to knot surface area (mm^2^), respectively [17]. Bacterial inoculation was performed in March 2023 by introducing bacterial suspensions (approximately 10^8^ CFU mL^−1^; McFarland 0.5) into bark wounds of 1–2-year-old olive stems, following the pathogenicity protocol described by Košćak et al. [17]. Initial symptoms appeared three months post-inoculation, with knot measurements conducted after six months, coinciding with most of the plants developing knots at the inoculation sites [17].

In the fungal experiment, ‘Istarska bjelica’ and ‘Buža’ were inoculated with *Dothiorella iberica* A.J.L. Phillips, J. Luque & A. Alves and *Neofusicoccum parvum* (Pennycook & Samuels) Crous, Slippers & A.J.L. Phillips, previously identified as the least and most aggressive fungal species (a total average necrotic lesion diameter of 4.46 and 93.45 mm), respectively [18]. Fungal inoculation was performed in December 2022 by inserting a 5-mm mycelial plug from a 10-day-old PDA culture into bark wounds measuring 5 mm in diameter, following Petrović et al. [18]. The first symptom observed on olive seedlings was leaf wilting, defoliation, and twig and branch dieback, which appeared after three months. Other symptoms that appeared included a change in bark color to reddish due to branch dieback, followed by the appearance of canker formations, bark cracking, and necrosis [18].

These pathogens were also selected based on their known pathogenic potential in olive [19,20]. Corresponding mock-inoculated controls were included for both experiments. In the bacterial experiment, control plants were wounded in the same manner as inoculated plants and treated with sterile distilled water. In the fungal experiment, control plants were wounded and treated with sterile PDA plugs without fungal mycelium. In each experiment, every cultivar × treatment combination comprised four biological replicates, with each replicate consisting of one olive seedling.

For simplicity, the cultivar × treatment combinations are abbreviated throughout the manuscript, tables, figures and Supplementary Material. In the bacterial experiment, IBB45, IBI7 and IBN denote ‘Istarska bjelica’ inoculated with isolate B45, isolate I7 and the corresponding control, respectively, whereas RB45, RI7 and RN denote the corresponding treatments for ‘Rosinjola’. In the fungal experiment, IBNP, IBDOI and IBN denote ‘Istarska bjelica’ inoculated with *Neofusicoccum parvum*, *Dothiorella iberica* and the corresponding control, respectively, whereas BNP, BDOI and BN denote the corresponding treatments for ‘Buža’.

### 2.2. Leaf Sampling and Sample Preparation

Olive leaves used for biochemical analyses were collected from experimental olive seedlings that had undergone bacterial and fungal inoculation treatments during greenhouse pathogenicity tests and the evaluation of variety resistance [17,18]. For the bacterial experiment, leaves were collected in April 2024 (4 plants/per strain/per cultivar), approximately 13 months after inoculation. For the fungal experiment, leaves were collected in November 2023 (4 plants/per fungal treatment/per cultivar), approximately 11 months after inoculation. The two experiments were sampled in different months (April and November), and seasonal variation in the composition of olive leaves is well documented [11]; the two datasets are therefore presented and interpreted separately. From each seedling, 40 fully expanded, mature leaves were sampled early in the morning, and immediately processed for metabolite analysis. Leaves were dried at 30 °C, ground to obtain a fine powder using a laboratory mill ZM200, Retsch GmbH, Haan, Germany) to ensure sample homogeneity, and stored at −20 °C until further analysis. Approximately 300 mg of finely ground, dry olive leaf powder was used for metabolite extraction. For metabolomic profiling, plant material was mixed with 10 mL of aqueous methanol (80%) by vortex and subsequently sonicated for 30 min in an ultrasound bath (DCG-250H, MRC Ltd. Holon, Israel). The extracts were macerated on an orbital shaker (GFL 3005, Lab Unlimited, Dublin, Ireland) at 25 °C and 150 rpm for 20 h. Afterwards, the extracts were centrifuged at 16,000× *g* for 10 min (Centric 350, Domel d.o.o., Železniki, Slovenia), filtered through a 0.22 µm nylon syringe filter, and stored frozen until further analysis. All analyses were conducted in one analytical batch.

### 2.3. Chemicals and Standards

Methanol (≥99.9%, HPLC grade), acetonitrile (≥99.9%, HPLC grade) and formic acid (≥99.9%, HPLC grade) used for chromatographic analyses were supplied by Merck (Darmstadt, Germany). Ultrapure deionized water was produced using a Siemens UltraClear purification system (Siemens AG, Munich, Germany). Analytical standards of appropriate purity were used for metabolite identification and quantification.

### 2.4. Determination of Primary Metabolites

Primary metabolite profiling was carried out via LC-MS/MS using a system comprising an autosampler (Nexera SIL-40CX3), two solvent delivery units (Nexera LC-40DX3), a thermostatted column compartment (Nexera CTO-40C), and an LCMS-8045 triple quadrupole mass spectrometer (all Shimadzu Corporation, Kyoto, Japan). Organic acid and amino acid separation was performed on a Discovery^®^ HS F5-3 core–shell column (2.1 mm × 150 mm, 3 µm; Sigma-Aldrich, St. Louis, MO, USA) at 37 °C, following the approach of Polić Pasković et al. [21]. Each 1 µL injection was resolved using a linear gradient of mobile phase A (water with 0.1% formic acid) and mobile phase B (acetonitrile with 0.1% formic acid) at a flow rate of 0.25 mL/min, programmed as follows: 100% A held from 0–2 min; decreasing to 75% A by 5 min; further decreasing to 65% A by 11 min; dropping to 5% A by 15 min; held at 5% A until 20 min; returning to 100% A by 20.1 min; and maintained at 100% A through 25 min.

For sugar quantification, a separate 1 µL injection was injected onto a 4.6 × 250 mm Asahipak NH2P-50 4E column (Shodex, Tokyo, Japan), with sugars resolved using a linear gradient of mobile phase A (water) and mobile phase B (acetonitrile): 20% A to 50% A over 0–20 min; 50% A to 25% A between 20–20.1 min; and held at 25% A from 20.1–25 min.

Individual sugars, organic acids, and amino acids were identified by comparing their retention times and precursor product pairs with those of corresponding analytical standards and quantified using external calibration curves (Supplementary Table S1). Organic acids and sugars were expressed as g kg^−1^ DW, whereas amino acids were expressed as mg kg^−1^ DW.

### 2.5. Determination of Lipid Peroxidation

Lipid peroxidation in olive leaves was estimated by measuring malondialdehyde (MDA), a secondary product of membrane lipid oxidation, using the thiobarbituric acid (TBA) assay according to the classical spectrophotometric assay described by Heath and Packer [22]. Briefly, approximately a 15 mg portion of previously dried sample was weighed out and homogenised in 1 mL of ice-cold 0.1% trichloroacetic acid (TCA) using a bead mill (Bead Raptor Elite, Omni International, Kennesaw, GA, USA) for 30 s at 4 m/s. The homogenate was then centrifuged (Centric 350, Domel d.o.o., Železniki, Slovenia) for 7 min at 16,000 g. A 400 µL aliquot of the resulting supernatant was combined with 1 mL of 0.5% thiobarbituric acid (TBA) prepared in 20% TCA. Reaction mixtures were incubated in a water bath at 95 °C for 30 min (GFL 1013, Gesellschaft für Labortechnik GmbH, Burgwedel, Germany), then rapidly cooled on ice. Following cooling, samples were centrifuged again for 7 min at 16,000 g, and the absorbance of the resulting supernatant was measured at both 600 nm and 532 nm.

MDA content was calculated according to the following equation: nmol MDA/g DW = A × 3.5 × 1000/(ε × b × y), where A represents the difference between absorbance at 532 nm and 600 nm; 3.5 is the dilution factor; 1000 converts µmol to nmol; ε is the molar extinction coefficient (155 mM^−1^cm^−1^); b is the pathlength (0.56 cm for a 200 µL volume); and y is the dry weight (g) of sample extracted. Final values are reported as nmol MDA/g DW. MDA was determined in oven-dried leaf powder because the same homogeneous material was used for all metabolite analyses. Although drying and storage may increase lipid oxidation, all samples from both experiments were dried, milled and stored under identical conditions for comparable periods. Therefore, any drying-related effect is expected to be consistent across treatments and cultivars and is unlikely to fully account for the observed differences.

### 2.6. Identification and Quantification of Phenolic Compounds

Phenolic compounds from olive leaves were analyzed with an LC-MS/MS system following a previously established protocol described by Žurga et al. [2,23], The LC-MS/MS system comprised an autosampler (Nexera SIL-40CX3), two solvent delivery units (Nexera LC-40DX3), and a thermostatted column compartment (Nexera CTO-40C), all interfaced with an LCMS-8045 triple quadrupole mass spectrometer (all Shimadzu Corporation, Kyoto, Japan). Separation was performed on an Agilent (Agilent Technologies, Palo Alto, CA, USA) C18 core–shell column (2.1 mm × 150 mm, 2.7 μm) maintained at a column temperature of 37 °C, with a 1 μL sample injection. A binary mobile phase system was used, consisting of water with 0.1% acetic acid (solvent A) and methanol with 0.1% acetic acid (solvent B), delivered at a constant flow rate of 0.35 mL/min. Separation was achieved via the following linear gradient: an isocratic hold at 98% A from 0 to 0.75 min; a gradual decrease to 50% A between 0.75 and 15 min; a further drop to 0% A from 15 to 15.1 min; a hold at 0% A until 20 min; a return to 98% A between 20 and 20.1 min; and re-equilibration at 98% A through 25 min. All analyzed metabolites were identified and quantified by comparing retention times, precursor/product ion transitions, and peak areas with those of corresponding analytical standards. Total analyzed phenolics were calculated as the sum of the concentrations of all individually quantified phenolic compounds (40 compounds in the bacterial experiment and 55 in the fungal experiment; Supplementary Tables S2 and S3) and are reported in the same units. This value is not equivalent to total phenolic content determined by spectrophotometric assays such as Folin–Ciocalteu, and the two should not be compared directly.

### 2.7. Statistical Analysis

Both experiments were arranged as completely randomized designs. The bacterial and fungal experiments included two olive cultivars (‘Istarska bjelica’ and ‘Rosinjola’—bacterial; ‘Istarska bjelica’ and ‘Buža’- fungal). Each cultivar × treatment combination comprised four independent biological replicates, each consisting of one olive plant (n = 4). Because individual analytes were occasionally below the limit of quantification in one or more replicates, the number of quantifiable observations per cell was lower than four for a subset of metabolites. Although this level of replication is limited, it is commonly used in controlled plant physiology and targeted metabolomics studies, and it is reported here per cell so that the precision of each estimate can be judged directly. All statistical analyses were performed using R (version 4.5.3) [24]. Two-way analysis of variance (ANOVA) and principal component analysis (PCA) were performed using functions from the stats package (version 4.5.3). Homogeneity of variances was assessed using Levene’s test implemented in the car package (version 3.1-5). Estimated marginal means together with Tukey’s post hoc comparisons were obtained using the emmeans package (version 2.0.3), whereas Dunnett’s test for comparisons with the corresponding controls was performed using the multcomp package (version 1.4-30). Graphical visualizations were prepared using the ggplot2 package (version 4.0.3), and heatmaps were generated using the pheatmap package (version 1.0.13). Results are presented as mean values ± standard errors (SE). Two-way analysis of variance (ANOVA) was selected because the experimental design included two fixed factors (cultivar and treatment) and their interaction, which directly addressed the objectives of the study.

The data were analyzed using two-way analysis of variance (ANOVA) to evaluate the effects of cultivar and treatment, including their interaction. In the bacterial experiment, the factors included cultivar and bacterial isolate, whereas in the fungal experiment the factors included cultivar and pathogen treatment. When significant effects were detected, Tukey’s post hoc test was used for pairwise comparisons among treatments at a significance level of *p* ≤ 0.05. Dunnett’s test was additionally applied to compare each treatment with the corresponding control. Each metabolite was treated as a separate response variable and constituted a separate family of hypotheses. Multiple comparisons within each metabolite were controlled using Tukey-adjusted pairwise comparisons and Dunnett-adjusted comparisons with the corresponding control. Therefore, no additional global false discovery rate (FDR) correction was applied across different metabolites. Given the number of metabolites analyzed, individual metabolite-level findings were interpreted cautiously and in the context of overall response patterns.

Analyte concentrations below the limit of quantification (LOQ; Supplementary Table S1) were reported as “<LOQ” and treated as missing values (NA); they were not replaced by zeros or imputed values. Post hoc comparisons were performed only for cultivar × treatment cells with at least two quantifiable replicates; cells with fewer observations are shown without a letter or asterisk. Two-way ANOVA was computed for all metabolites for which at least one quantifiable replicate was available in every cultivar and in every treatment level, so that both main effects could be estimated. For ten metabolites, one or more cells contained no quantifiable replicate or only a single one (Aspartic acid and Glutamine in the bacterial experiment; Succinic acid, Aspartic acid, Alanine, Biotin, Glutamine, Histidine, Lysine and Tryptophan in the fungal experiment); for these metabolites the ANOVA is unbalanced, and the corresponding *p*-values should be interpreted with caution.

Principal component analysis (PCA) was performed using the prcomp function from the stats package on standardized data (zero mean and unit variance) based on the correlation matrix. PCA was used as an exploratory multivariate method to visualize patterns of metabolite variation among samples and should therefore be interpreted as a descriptive rather than an inferential analysis. Principal component analysis was performed on all primary metabolites quantified in the respective experiment. PCA was used to visualize sample distribution according to cultivar and bacterial isolate or fungal pathogen treatment and to identify the metabolites contributing most strongly to sample separation. For graphical clarity, only the metabolites with the largest loading vectors in the PC1–PC2 plane were labelled in the PCA biplots, whereas complete PCA scores and loadings for all included metabolites are provided as Supplementary Material.

Heatmap analysis was performed for phenolic compounds using the pheatmap package. Prior to visualization, metabolite concentrations were standardized using z-scores. Heatmaps were constructed from treatment means, and hierarchical clustering was performed using Euclidean distance based on treatment means.

The raw biological replicate data used for all statistical analyses are provided as Supplementary Data S1.

## 3. Results

The metabolic responses of olive leaves were evaluated in two independent experiments: a bacterial inoculation experiment and a fungal pathogen experiment. Changes in primary metabolites, phenolic compounds and lipid peroxidation were analyzed within each experimental system to identify treatment- and cultivar-related differences. Both univariate statistical analyses and multivariate approaches were used to identify metabolites contributing most strongly to the observed variation among treatments. The following sections describe changes in primary metabolites, phenolic compounds, and lipid peroxidation in olive leaves exposed to bacterial isolates and fungal pathogens.

### 3.1. Primary Metabolites

Primary metabolite profiles were analyzed separately for the bacterial and fungal experiments to evaluate treatment-related changes in sugars, organic acids and free amino acids. Principal component analysis (PCA) was used to provide an integrated overview of metabolite variation and to identify compounds contributing most strongly to differences among treatments.

#### 3.1.1. Response to Bacterial Isolates

Primary metabolite profiles differed among bacterial treatments and between the two olive cultivars (Table 1). Several metabolites were significantly influenced by cultivar, isolate or their interaction.

Among sugars, glucose concentrations ranged from 5.81 to 10.27 g kg^−1^ DW, with the lowest values observed in ‘Istarska bjelica’ inoculated with isolate I7 and the highest in ‘Rosinjola’ treated with the same isolate. Two-way ANOVA indicated significant effects of cultivar, isolate and their interaction (*p* < 0.001). Fructose concentrations varied between 3.92 and 5.80 g kg^−1^ DW, showing significant effects of both cultivar (*p* = 0.006) and isolate (*p* = 0.020). Sucrose showed the largest variation among sugars, ranging from 40.53 to 86.78 g kg^−1^ DW, with generally higher concentrations in ‘Istarska bjelica’, particularly after inoculation with isolate B45, whereas lower concentrations were detected in ‘Rosinjola’ following treatment with isolate I7. Mannitol concentrations were comparatively stable, varying between 30.65 and 40.16 g kg^−1^ DW, and were significantly affected only by cultivar (*p* < 0.001), while isolate treatment had no significant effect.

Among organic acids, ascorbic acid concentrations ranged from 0.77 to 1.72 g kg^−1^ DW and were significantly influenced by isolate treatment (*p* = 0.022). In contrast, malic and quinic acids showed relatively limited variation across treatments and were not significantly affected by cultivar or isolate.

Free amino acids displayed the greatest treatment-related variation. Tryptophan showed the largest variability among the detected metabolites, ranging from 49.58 to 367.42 mg kg^−1^ DW, with particularly high concentrations in samples inoculated with isolate I7. Both cultivar and isolate significantly influenced tryptophan accumulation (*p* < 0.001). Glutamic acid concentrations ranged from 9.50 to 25.08 mg kg^−1^ DW and were significantly affected by isolate (*p* < 0.001) as well as by the cultivar × isolate interaction (*p* = 0.001). Lysine concentrations were significantly affected by isolate (*p* = 0.045), while aspartic acid showed significant effects of isolate (*p* = 0.013) and the cultivar × isolate interaction (*p* = 0.004).

**Figure 1:**
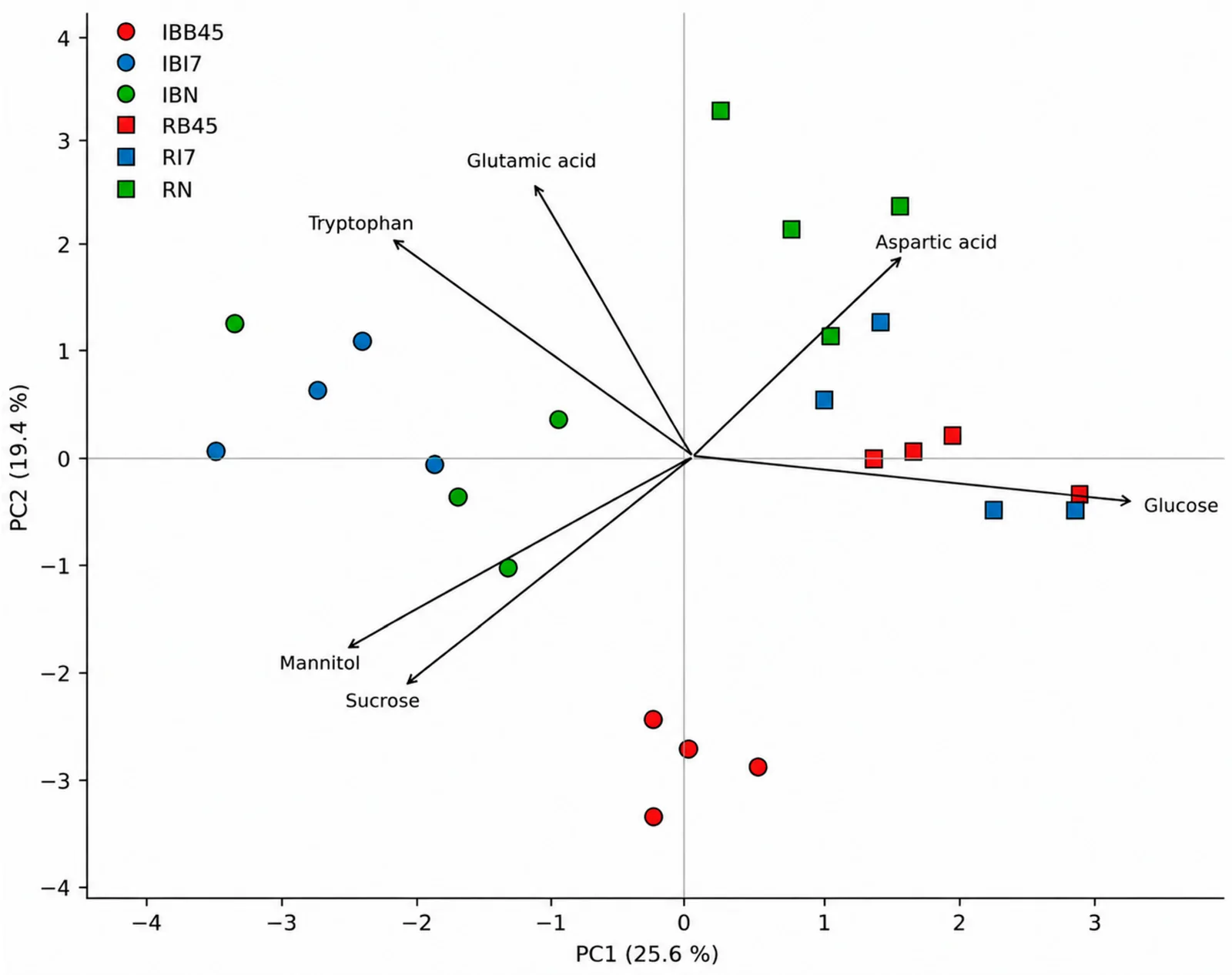
Principal component analysis (PCA) of primary metabolites detected in olive leaves following inoculation with bacterial isolates. Each point represents an individual biological replicate. Samples are identified according to cultivar and treatment combination: IBB45 = ‘Istarska bjelica’ inoculated with isolate B45, IBI7 = ‘Istarska bjelica’ inoculated with isolate I7, IBN = ‘Istarska bjelica’ negative control, RB45 = ‘Rosinjola’ inoculated with isolate B45, RI7 = ‘Rosinjola’ inoculated with isolate I7 and RN = ‘Rosinjola’ negative control. Circle symbols represent ‘Istarska bjelica’, whereas squares represent ‘Rosinjola’. Colors indicate bacterial treatments (red = B45, blue = I7, green = negative control). Arrows indicate the six metabolites with the greatest loading-vector length in the PC1–PC2 plane. Percentages shown on the axes represent the proportion of total variance explained by the first two principal components.

Principal component analysis (PCA) further illustrated the differences in primary metabolite profiles among treatments (Supplementary Tables S4 and S5; Figure 1). The first two principal components explained 45.0% of the total variance (PC1 = 25.6%, PC2 = 19.4%). Samples showed partial separation according to both bacterial isolate treatment and cultivar. Separation along PC1 was mainly associated with sugars, particularly glucose, which showed strong positive loadings and contributed to the positioning of samples on the right side of the ordination space. In contrast, several amino acids, including tryptophan and glutamic acid, were more strongly associated with PC2, contributing to the vertical separation of treatments. Mannitol and sucrose were positioned opposite to glucose along PC1. Overall, sugar and amino acids contributed most strongly to the separation of samples in the PCA ordination.

#### 3.1.2. Response to Fungal Pathogens

Primary metabolite profiles differed among fungal pathogen treatments, although the magnitude of responses varied among metabolite groups and between cultivars (Table 2). While sugars exhibited relatively small treatment-related differences, several organic acids and particularly free amino acids showed more pronounced responses to pathogen inoculation.

Among sugars, overall variation among treatments remained relatively limited. Glucose concentrations ranged from approximately 6.5 to 26.6 g kg^−1^ DW across treatments but were not significantly influenced by cultivar or pathogen treatment. Fructose concentrations varied between approximately 1.2 and 4.7 g kg^−1^ DW and were significantly affected by the cultivar × pathogen interaction. Sucrose concentrations ranged from about 18.6 to 26.5 g kg^−1^ DW and differed significantly between cultivars, as well as in the cultivar × pathogen interaction, reflecting a significant interaction.

In the group of organic acids, most compounds remained relatively stable across treatments. Citric acid concentrations ranged from approximately 0.17 to 1.06 g kg^−1^ DW and were significantly influenced by pathogen treatment as well as by the cultivar × pathogen interaction. Similarly, quinic acid varied between approximately 9.95 and 16.47 g kg^−1^ DW and showed significant pathogen and interaction effects. Succinic acid responded strongly to pathogen treatment, whereas ascorbic acid (0.27 to 0.46 g kg^−1^ DW) and malic acid (2.33 to 3.80 g kg^−1^ DW) did not show significant changes associated with the tested factors.

The greatest variability was observed among free amino acids, indicating that this metabolite group showed the largest treatment-related variation following fungal inoculation. Several amino acids were significantly affected by cultivar, including aspartic acid, phenylalanine, serine, and tryptophan. Tryptophan concentrations showed particularly large variation, ranging from approximately 92 to 393 mg kg^−1^ DW depending on cultivar and treatment. Pathogen treatment significantly influenced histidine, glutamic acid, phenylalanine, proline and serine. In addition, significant cultivar × pathogen interactions were detected for several metabolites, including glutamic acid, phenylalanine and proline. Some metabolites were not detected in all treatments or cultivars and therefore appear as missing values (NA) in the dataset.

**Figure 2:**
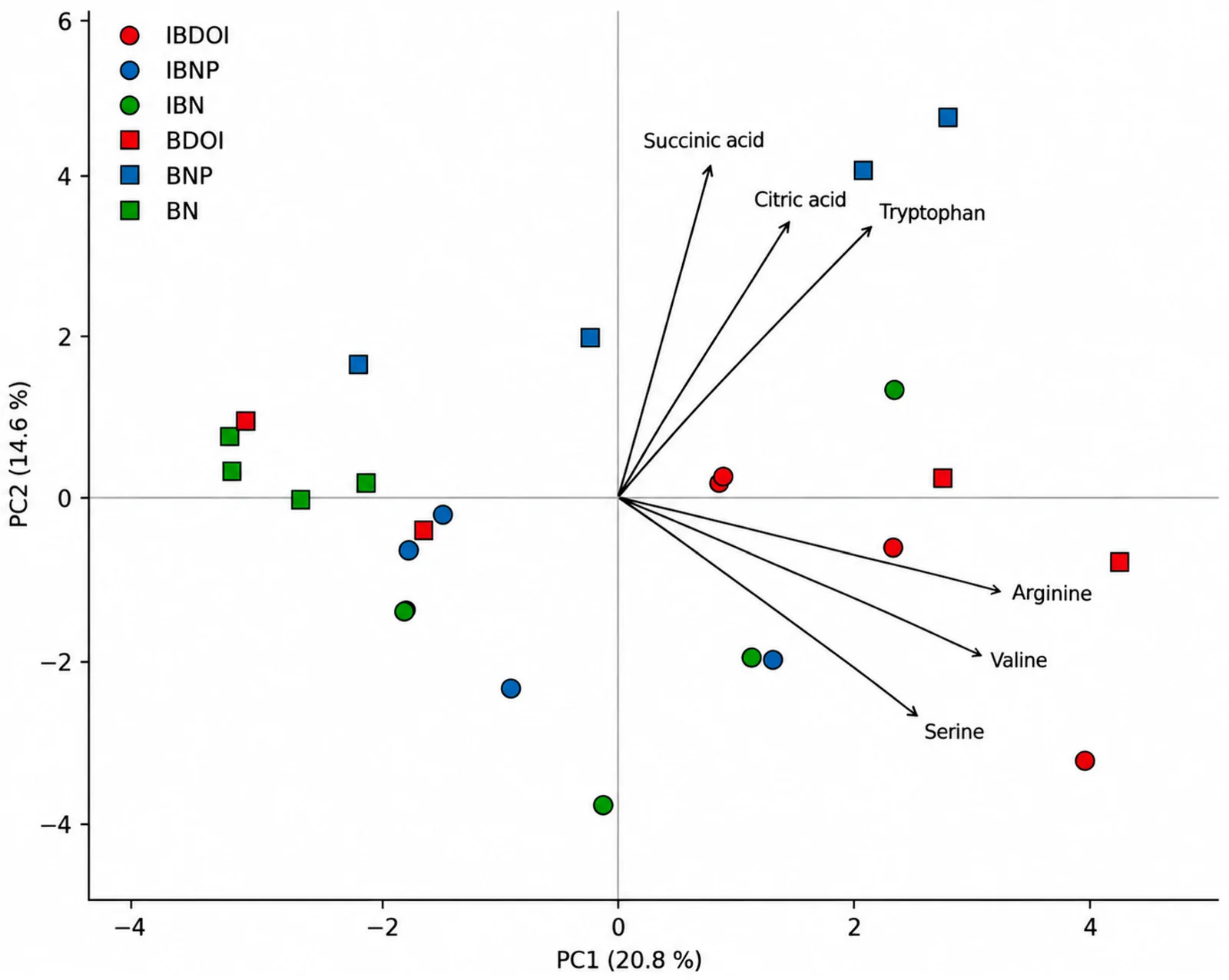
Principal component analysis (PCA) of primary metabolites detected in olive leaves following inoculation with fungal pathogens. Each point represents an individual biological replicate. Samples are identified according to cultivar and treatment combinations (IBDOI = ‘Istarska bjelica’ inoculated with *Dothiorella iberica*, IBNP = ‘Istarska bjelica’ inoculated with *Neofusicoccum parvum*, IBN = ‘Istarska bjelica’ negative control, BDOI = ‘Buža’ inoculated with *Dothiorella iberica*, BNP = ‘Buža’ inoculated with *Neofusicoccum parvum* and BN = ‘Buža’ negative control). Circle symbols represent ‘Istarska bjelica’, whereas squares represent ‘Buža’. Colors indicate pathogen treatments (red = *Dothiorella iberica*, blue = *Neofusicoccum parvum*, green = negative control). Arrows indicate the six metabolites with the greatest loading-vector length in the PC1–PC2 plane. Percentages shown on the axes represent the proportion of total variance explained by the first two principal components.

The principal component analysis (PCA) further illustrated these patterns (Supplementary Tables S6 and S7; Figure 2). The first two principal components explained 35.4% of the total variance (PC1 = 20.8%, PC2 = 14.6%). Several amino acids, particularly arginine, valine and serine, contributed strongly to the separation of samples along PC1, whereas organic acids, especially succinic and citric acid, were more strongly associated with PC2. The distribution of samples indicated partial grouping according to both pathogen treatment and cultivar. A greater dispersion of samples was observed for cultivar ‘Buža’

### 3.2. Phenolic Compounds

#### 3.2.1. Response of Phenolic Compounds to Bacterial Isolates

**Figure 3:**
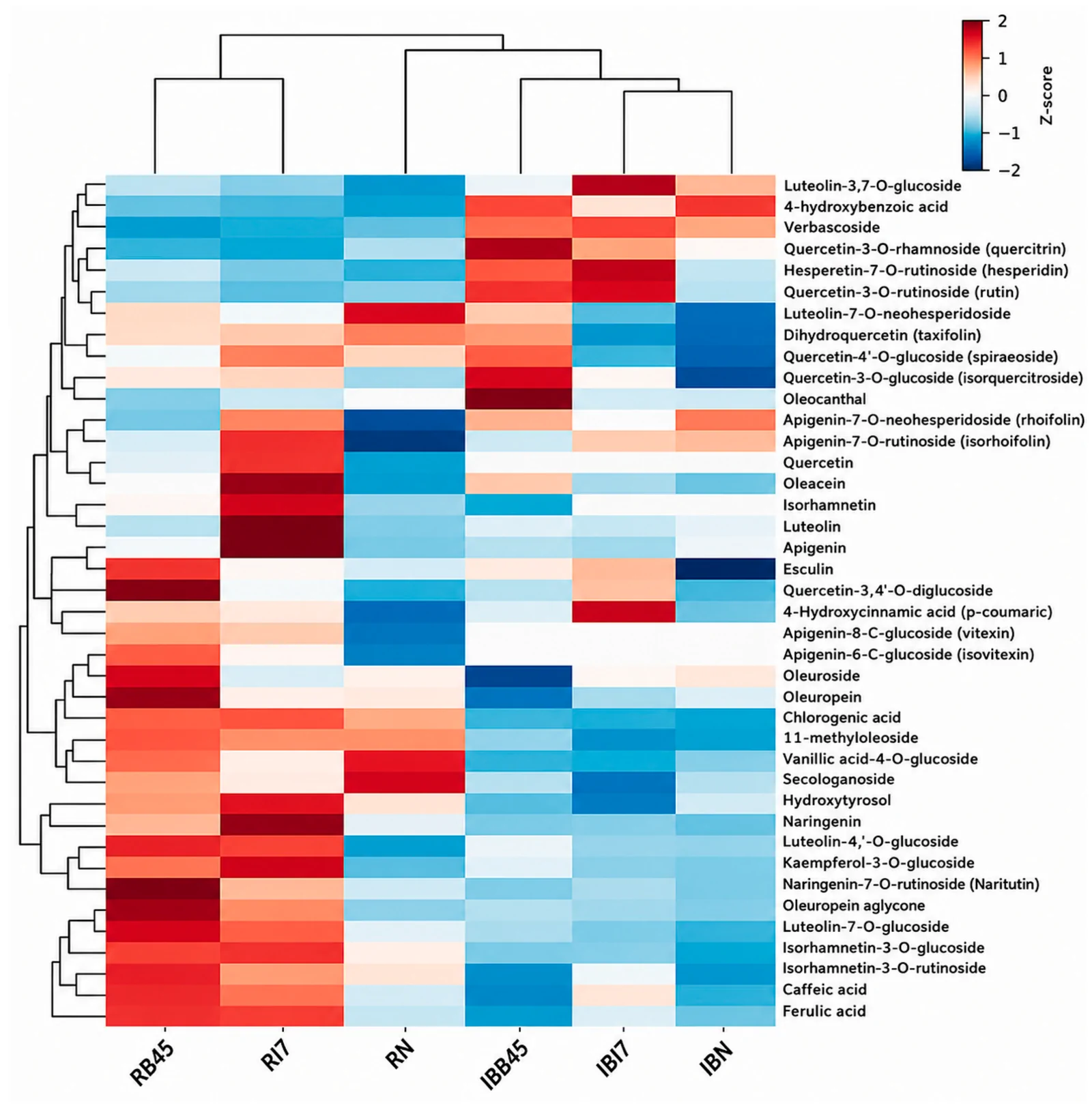
Heatmap showing the relative abundance of phenolic compounds detected in olive leaves following inoculation with bacterial isolates. Rows correspond to individual phenolic compounds and columns represent treatment combinations: IBB45 (‘Istarska bjelica’ + isolate B45), IBI7 (‘Istarska bjelica’ + isolate I7), IBN (‘Istarska bjelica’ negative control), RB45 (‘Rosinjola’ + isolate B45), RI7 (‘Rosinjola’ + isolate I7) and RN (‘Rosinjola’ negative control). Color intensity reflects standardized values (z-scores) of phenolic concentrations. Hierarchical clustering of both treatments and phenolic compounds was performed using treatment means and Euclidean distance.

Phenolic profiles differed among bacterial treatments (Supplementary Table S2; Figure 3). Overall, a number of phenolic compounds exhibited significant effects of cultivar, isolate and their interaction, indicating cultivar-specific metabolic responses to bacterial challenge.

Several compounds showed significant differences to bacterial inoculation. For example, caffeic acid and p-coumaric acid showed strong isolate effects (*p* < 0.001), while higher concentrations of ferulic acid were observed in leaves inoculated with bacterial isolates compared with controls. Chlorogenic acid was strongly influenced by cultivar (*p* < 0.001) and showed moderate isolate effects.

Pronounced differences were also observed among flavonoids. Several flavonoid glycosides, including luteolin-3,7-*O*-glucoside, quercetin-3-*O*-rutinoside (rutin), and quercetin-3-*O*-glucoside, showed significant isolate-dependent variation. In particular, rutin and related quercetin derivatives showed higher concentrations in leaves inoculated with bacterial isolates than in control treatments. Other flavonoids, such as hesperetin-7-*O*-rutinoside (hesperidin) and quercetin-3-*O*-rhamnoside, were significantly affected by both cultivar and isolate factors.

Compounds belonging to the secoiridoid group, which represent characteristic phenolics of olive leaves, also exhibited notable variation. Secoiridoid oleuropein, the most abundant compound detected in olive leaves, ranged from approximately 27.9 to 38.3 g kg^−1^ DW across treatments (Supplementary Table S2). Oleuropein differed significantly between cultivars (*p* < 0.001) and showed cultivar-dependent responses to bacterial inoculation. Similarly, oleuropein aglycone, oleacein and oleuroside showed significant cultivar or isolate effects.

Consistent with these compound-specific responses, the total analysed phenolic content also differed significantly among treatments, with significant effects of cultivar (*p* < 0.001), isolate (*p* = 0.003) and their interaction (*p* < 0.001).

The heatmap and hierarchical clustering analysis further illustrated these patterns (Figure 3). Phenolic compounds were grouped according to similarities in their accumulation profiles across treatments. The heatmap revealed cultivar- and isolate-related differences in phenolic accumulation patterns.

#### 3.2.2. Response of Phenolic Compounds to Fungal Inoculation

**Figure 4:**
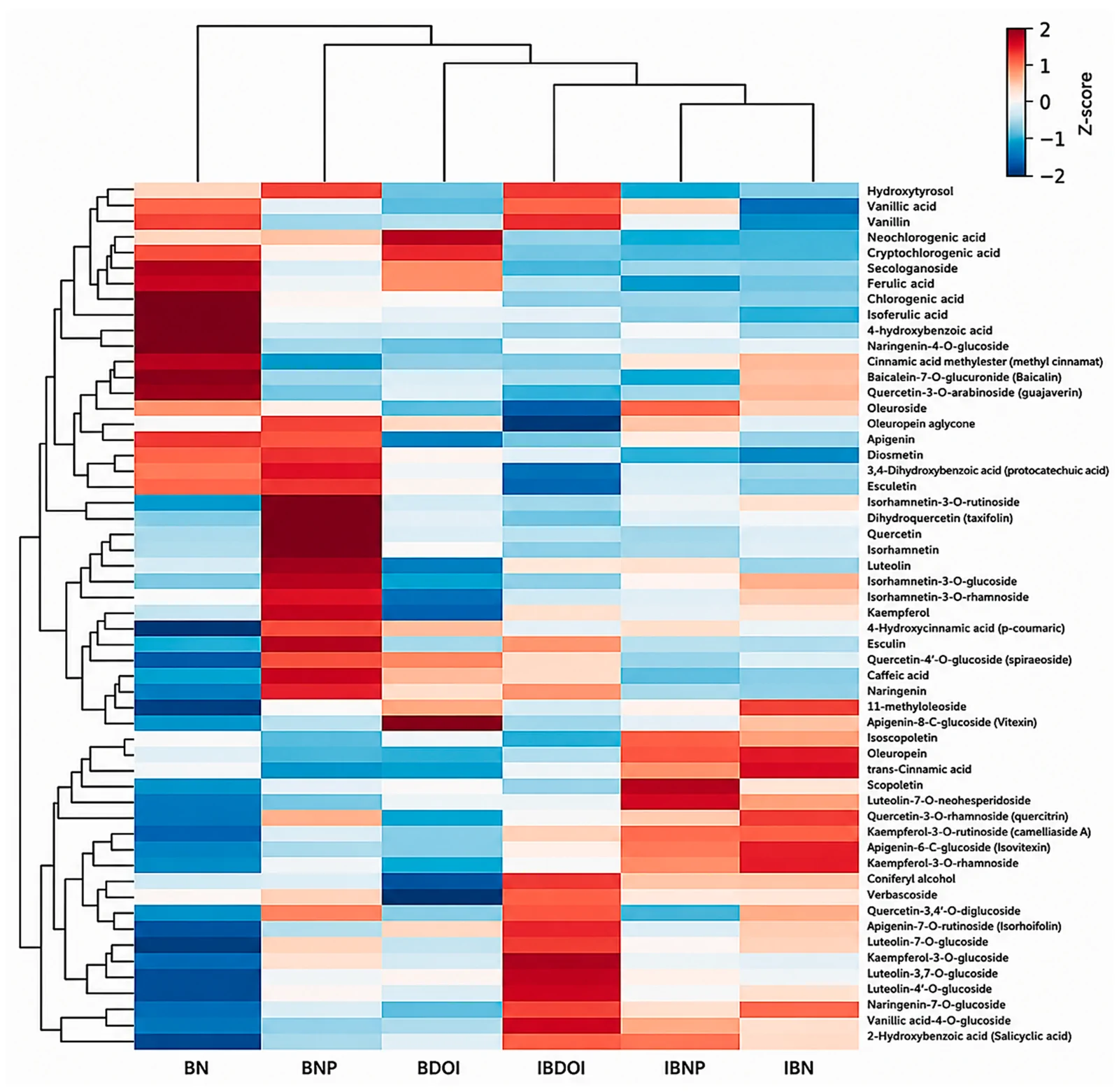
Heatmap representing the variation in phenolic compounds detected in olive leaves following inoculation with the fungal pathogens *Dothiorella iberica* and *Neofusicoccum parvum*. Rows correspond to individual phenolic compounds and columns represent treatment combinations: IBDOI (‘Istarska bjelica’ + pathogen *Dothiorella iberica*), IBNP (‘Istarska bjelica’ + pathogen *Neofusicoccum parvum*), IBN (‘Istarska bjelica’ negative control), BDOI (‘Buža’ + pathogen *Dothiorella iberica*), BNP (‘Buža’ + pathogen *Neofusicoccum parvum*) and BN (‘Buža’ negative control). Color gradients indicate standardized phenolic concentrations (z-scores), highlighting relative increases or decreases across treatments. Hierarchical clustering of both treatments and phenolic compounds was performed using treatment means and Euclidean distance.

Phenolic profiles differed among fungal pathogen treatments (Supplementary Table S3; Figure 4). Several phenolics showed significant effects of cultivar, pathogen treatment or their interaction, indicating cultivar-specific metabolic responses to fungal inoculation.

Among phenolic acids, some compounds responded significantly to pathogen treatment. For example, p-coumaric acid showed a strong pathogen effect (*p* = 0.002) and a significant cultivar × pathogen interaction, with concentrations ranging from approximately 0.53 to 6.29 mg kg^−1^ DW across treatments. Similarly, chlorogenic acid was strongly influenced by cultivar and pathogen treatment (*p* < 0.001), varying between approximately 14.7 and 142.8 mg kg^−1^ DW and with generally higher concentrations detected in ‘Buža’. Other acids, including ferulic acid and isoferulic acid, also exhibited significant cultivar-related differences.

Pronounced variation was also observed among flavonoids. Several flavonoid glycosides, including luteolin-3,7-*O*-glucoside, luteolin-7-*O*-glucoside and quercetin derivatives, were significantly affected by cultivar and pathogen treatment. In particular, luteolin-7-*O*-glucoside concentrations ranged from approximately 1339 to 1997 mg kg^−1^ DW depending on cultivar and pathogen treatment. Compounds belonging to the quercetin and kaempferol families showed significant pathogen and/or cultivar × pathogen interaction effects.

Compounds belonging to the secoiridoid group, characteristic metabolites of olive tissues, showed comparatively smaller treatment-related changes. Major secoiridoids such as oleuropein and oleuroside did not differ significantly among pathogen treatments, with oleuropein concentrations remaining relatively stable between 29.7 and 32.6 g kg^−1^ DW. Similarly, the total analysed phenolic content did not differ significantly among treatments.

The heatmap and hierarchical clustering analysis further illustrated variation in phenolic profiles among samples (Figure 4). Phenolic compounds were grouped according to similarities in their accumulation patterns, revealing both cultivar-related and pathogen-associated variation.

### 3.3. Lipid Peroxidation

**Figure 5:**
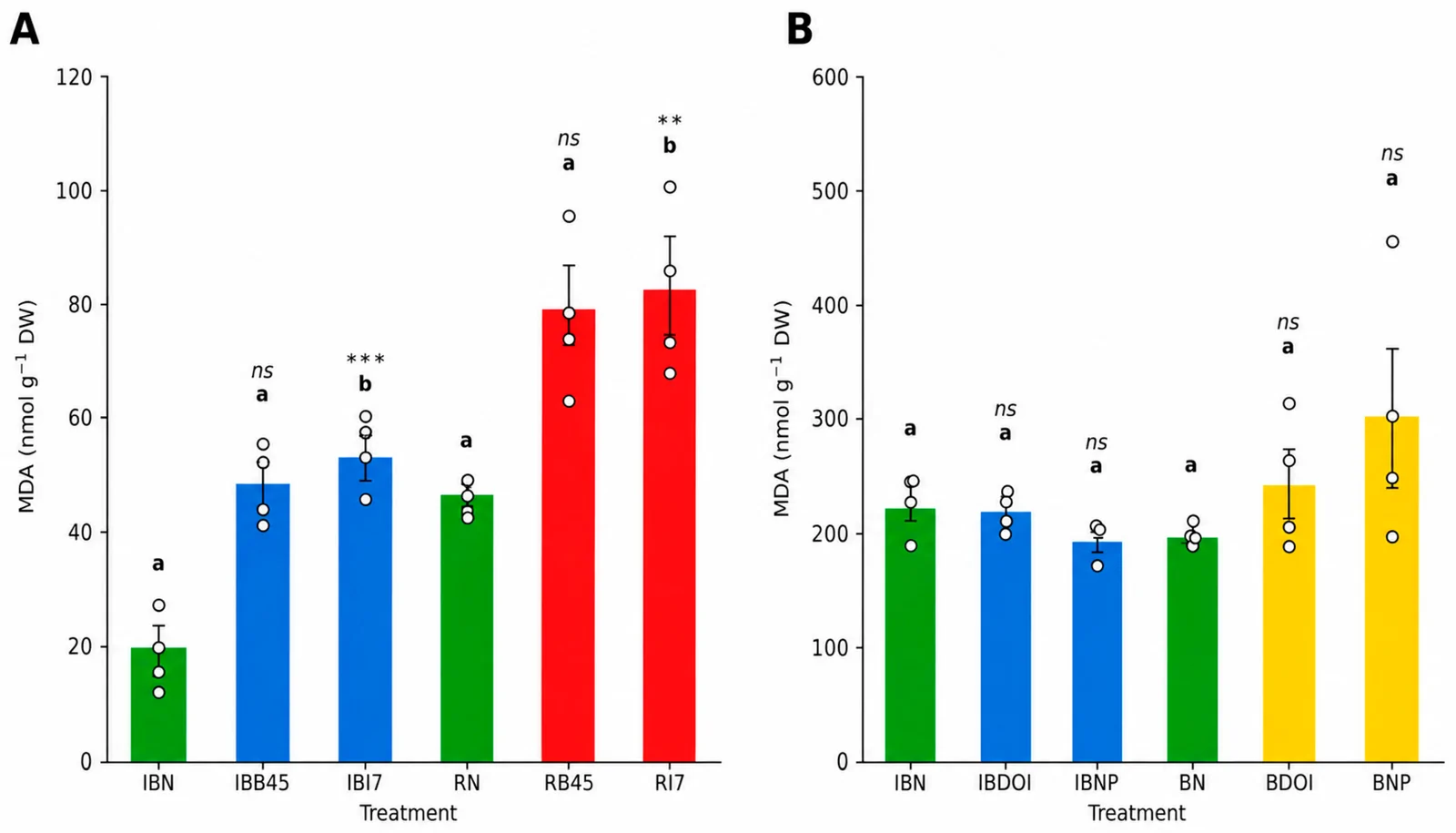
Lipid peroxidation in olive leaves expressed as malondialdehyde (MDA) concentration (nmol g^−1^ DW) following bacterial inoculation or fungal inoculation. (**A**) Effects of bacterial isolates (B45 and I7) on MDA levels in the cultivars ‘Istarska bjelica’ and ‘Rosinjola’. Treatments are coded as follows: IBB45 (‘Istarska bjelica’ + B45), IBI7 (‘Istarska bjelica’ + I7), IBN (‘Istarska bjelica’ negative control), RB45 (‘Rosinjola’ + B45), RI7 (‘Rosinjola’ + I7), and RN (‘Rosinjola’ negative control). (**B**) Effects of the fungal pathogens *Dothiorella iberica* and *Neofusicoccum parvum* on MDA levels in the cultivars ‘Istarska bjelica’ and ‘Buža’. Treatments are coded as follows: IBDOI (‘Istarska bjelica’ + *Dothiorella iberica*), IBNP (‘Istarska bjelica’ + *Neofusicoccum parvum*), IBN (‘Istarska bjelica’ negative control), BDOI (‘Buža’ + *Dothiorella iberica*), BNP (‘Buža’ + *Neofusicoccum parvum*), and BN (‘Buža’ negative control). Bars represent mean values ± standard error (SE), and open circles indicate individual biological replicates. Different lowercase letters indicate significant differences among treatments according to Tukey’s HSD test (*p* ≤ 0.05). Asterisks indicate significant differences compared with the corresponding control according to Dunnett’s test (** *p* ≤ 0.01, *** *p* ≤ 0.001; ns = not significant).

Lipid peroxidation, expressed as malondialdehyde (MDA) concentration, was evaluated separately for the bacterial and fungal experiments (Figure 5).

In the bacterial experiment (Figure 5A), two-way ANOVA revealed significant effects of cultivar (*p* < 0.001) and isolate (*p* < 0.001), whereas their interaction was not significant. MDA concentrations ranged from approximately 20.3 to 82.6 nmol MDA g^−1^ DW. Inoculation with isolate I7 was associated with significantly higher MDA concentrations compared with the corresponding controls in both cultivars, whereas treatment with isolate B45 did not significantly affect MDA concentrations. In addition, the cultivar ‘Rosinjola’ exhibited higher MDA concentrations than ‘Istarska bjelica’, irrespective of bacterial treatment.

In contrast, fungal pathogen (Figure 5B) inoculation did not significantly affect lipid peroxidation. MDA concentrations in the fungal experiment ranged from approximately 190 to 301 nmol MDA g^−1^ DW. Neither cultivar (*p* = 0.113) nor pathogen treatment (*p* = 0.342) showed significant effects on MDA concentrations, although a marginal cultivar × pathogen interaction was observed (*p* = 0.058). Despite numerical variation among treatments, MDA concentrations did not differ significantly among fungal pathogen treatments (Figure 5B).

## 4. Discussion

### 4.1. Primary Metabolite Responses of Olive Leaves to Bacterial and Fungal Challenge

Primary metabolites are essential components of plant metabolism and are frequently affected during plant–microbe interactions. Metabolomic studies in olive have demonstrated that sugars, organic acids and amino acids constitute significant components of the olive metabolome, exhibiting considerable variability in response to genotype, environmental conditions and stress factors [2,7,25]. These metabolites provide metabolic energy and biosynthetic precursors required for plant growth and stress responses [3,4].

Carbohydrate metabolism showed treatment-related differences in the bacterial experiment. Among the sugars, sucrose exhibited the most significant variation, ranging from approximately 40 to 87 g kg^−1^ DW across treatments. The highest concentrations were observed in ‘Istarska bjelica’, where sucrose levels were approximately 114% higher than those measured in ‘Rosinjola’ under RI7 treatment. Such cultivar-dependent differences in carbohydrate metabolism are consistent with previous studies demonstrating that olive metabolic profiles are strongly influenced by genotype and physiological status [2,7]. Sugars have also been shown to function as signaling molecules, regulating plant defense responses and carbon allocation during the course of pathogen infection [3,26]. Similarly, fungi from the Botryosphaeriaceae family have been reported to interfere with sugar signaling and suppress the expression of genes related to plant immunity [27].

Glucose concentrations ranged from approximately 5.8 to 10.3 g kg^−1^ DW, with the highest values recorded in ‘Rosinjola’. The glucose levels in ‘Rosinjola’ were found to be approximately 77% higher than those observed in ‘Istarska bjelica’ under the I7 treatment. Although the variation was smaller than that observed for sucrose, glucose was also significantly affected by cultivar and bacterial isolate. Similar shifts in carbohydrate metabolism have been documented in metabolomic analyses of olive tissues, where sugars constitute one of the predominant metabolite classes identified in xylem sap and leaf extracts [2,7].

Treatment-related changes were more pronounced in amino acid metabolism. It is notable that tryptophan demonstrated the most significant variation among all the primary metabolites that were detected. In the ‘Istarska bjelica’ samples, tryptophan concentrations ranged from approximately 50 mg kg^−1^ DW in the B45 treatment to more than 360 mg kg^−1^ DW in the I7 treatment, representing an increase of approximately 600% relative to the lowest observed values. In ‘Rosinjola’, tryptophan levels ranged from approximately 96 to 229 mg kg^−1^ DW, indicating substantial metabolic variability across treatments. Amino acids are important intermediates linking primary metabolism with the synthesis of specialized metabolites involved in plant defense [5,28]. Tryptophan is an aromatic amino acid synthesized through the shikimate pathway and serves as a precursor for numerous secondary metabolites in plants [5,29]. The plant pathogenic bacterium *Pseudomonas savastanoi*, uses the so-called “indole-3-acetamide pathway” to convert tryptophan to indole-3-acetic acid (IAA), the phytohormone regulating the pathogenicity mechanisms, via a two-step pathway catalyzed by enzymes encoded by the genes in the iaaM/iaaH operon [30]. Moreover, pathovar *nerii* of *P. savastanoi* is able to conjugate IAA to lysine to generate the less biologically active compound IAA-Lys via the enzyme IAA-lysine synthase encoded by the iaaL gene. Interestingly, iaaL is now known to be widespread in many *Pseudomonas syringae* pathovars, even in the absence of the iaaM and iaaH genes for IAA biosynthesis [30,31]. Therefore, the observed variation in tryptophan concentrations is consistent with metabolic adjustments reported during plant responses to microbial challenge, although the present data do not allow conclusions regarding activation of this pathway.

Glutamic acid also showed considerable variation among treatments, with concentrations ranging from approximately 9.5 to 25.1 mg kg^−1^ DW, corresponding to an increase of about 164% between the lowest and highest observed values, indicating significant effects of bacterial isolate and the cultivar × isolate interaction. Glutamate and related amino acids play an important role in plant nitrogen metabolism and may participate in metabolic signaling processes during stress responses [6].

In the fungal experiment, amino acids showed greater variability than sugars and organic acids. This observation is consistent with previous studies reporting coordinated changes in amino acid metabolism during plant responses to stress [28,32]. Although the magnitude of metabolite responses differed among cultivars and pathogen treatments, amino acids represented the metabolite group showing the greatest variation [4,5].

Taken together, the bacterial and fungal experiments revealed different patterns of primary metabolite variation. In the bacterial experiment, differences were most evident in carbohydrate metabolism and selected amino acids, particularly tryptophan, whereas in the fungal experiment greater variation was observed among amino acids. Because the two experiments differed in plant material, pathogens and experimental conditions, these patterns should be interpreted within each experimental system rather than as direct quantitative comparisons. These observations are consistent with previous reports describing metabolic adjustments during plant responses to microbial challenge [3,4]. These observations are also supported in the PCA, in which sugars and amino acids contributed most strongly to the separation of treatments.

### 4.2. Phenolic Compounds and Cultivar-Dependent Phenolic Responses

Phenolic compounds are a major group of secondary metabolites involved in plant responses to biotic and abiotic stress. In olive, leaves are particularly rich in phenolic compounds, including secoiridoids, flavonoids and phenolic alcohols, which contribute to the antioxidant capacity and biological activity of olive tissues [2,8,33]. These compounds have frequently been associated with plant responses to biotic and abiotic stress and are considered important components of the olive secondary metabolome [34].

In the present study, secoiridoids represented the most abundant phenolic class detected in olive leaves. In particular, oleuropein concentrations ranged from approximately 27.9 to 38.3 g kg^−1^ DW depending on cultivar and treatment, consistent with its role as the dominant phenolic compound in olive leaves. This relatively wide range, corresponding to nearly 40% variation among treatments, highlights considerable variation in secoiridoid accumulation among cultivars and bacterial treatments. Previous studies have consistently reported that oleuropein represents the major phenolic constituent of olive leaves and is largely responsible for their antioxidant and antimicrobial properties [1,2,33].

Bacterial treatments were also associated with coordinated changes in oleuropein, oleuropein aglycone, oleacein and oleuroside. Similar relationships among secoiridoid derivatives have been reported in olive tissues under different environmental and physiological conditions [2,9]. Although the present data do not allow conclusions regarding metabolic fluxes, the coordinated variation among these metabolites is consistent with changes in the secoiridoid profile following bacterial challenge.

In contrast, the fungal experiment showed comparatively limited variation in the major secoiridoids despite changes observed in amino acid metabolism. One possible explanation is that oleuropein and related secoiridoids are constitutively abundant in olive leaves, such that fungal challenge may not necessarily result in further increases in their concentrations [10]. Previous studies have also suggested that fungal inoculation may influence the composition or relative proportions of secoiridoid derivatives rather than uniformly altering the concentrations of the major compounds [35]. However, because the present study did not investigate phenolic biosynthetic pathways or pathogen virulence mechanisms, these explanations remain hypothetical and require further investigation [36].

In addition to secoiridoids, several flavonoid derivatives were detected in olive leaves, including luteolin and apigenin glycosides. Flavonoids are widely recognized for their antioxidant activity and their potential contribution to protection against oxidative stress associated with pathogen challenge [2,14,16,34]. Accumulation of flavonoids and secoiridoids has frequently been reported during plant responses to microbial infection, where these compounds may contribute to antimicrobial activity as well as protection against oxidative damage associated with pathogen-induced stress [2,8].

Strong cultivar-dependent variation was also observed in phenolic composition. Total analysed phenolic concentrations in ‘Rosinjola’ exceeded 50 g kg^−1^ DW in RB45, whereas values in ‘Istarska bjelica’ remained below approximately 45 g kg^−1^ DW. Similar cultivar-dependent differences have been widely reported in olive, reflecting the influence of genotype on phenolic composition [2,12,13].

### 4.3. Oxidative Stress and Lipid Peroxidation Responses

Lipid peroxidation represents a common physiological indicator of oxidative stress in plant tissues and is frequently evaluated through the accumulation of malondialdehyde (MDA), a product of membrane lipid degradation. Increased MDA concentrations are typically associated with enhanced production of reactive oxygen species (ROS) during plant responses to environmental or biological stress [14,16]. In olive plants, oxidative stress responses have been reported under various stress conditions, including pathogen infection and abiotic stress [37].

The bacterial and fungal experiments differed in the observed patterns of MDA variation. In the bacterial experiment, MDA concentrations ranged from approximately 20 to 83 nmol MDA g^−1^ DW. Inoculation with isolate I7 resulted in significantly higher MDA concentrations than the corresponding controls in both cultivars, whereas isolate B45 did not significantly affect MDA concentrations. In addition, ‘Rosinjola’ consistently exhibited higher MDA concentrations than ‘Istarska bjelica’, indicating cultivar-dependent differences in oxidative responses following bacterial challenge.

In the fungal experiment, MDA concentrations ranged from approximately 190 to 301 nmol MDA g^−1^ DW. Despite numerical differences among treatments, neither cultivar nor pathogen treatment significantly affected MDA concentrations. Lipid peroxidation has previously been reported as a physiological response during plant–pathogen interactions [16,37]. Because no significant treatment effects were detected in the present experiment, these results suggest that oxidative status remained broadly similar among the fungal treatments.

Although only MDA was determined in the present study, the observed responses are consistent with previous reports identifying lipid peroxidation as a common physiological response during plant–pathogen interactions [14,15,16,37]. Additional measurements of antioxidant enzymes and reactive oxygen species would, however, be required to provide a more comprehensive assessment of oxidative stress responses. Because disease severity and additional oxidative stress markers were not assessed, the physiological significance of the observed MDA responses should be interpreted with caution. A further limitation is that MDA was measured in dried leaf powder, so the absolute values cannot be directly compared with studies using fresh tissue.

### 4.4. Integrated Metabolic Responses to Microbial Challenge

In a *Pss* study, the longitudinal sections of the collapsed stem portions revealed an extensive production of polysaccharide and phenolic substances that had accumulated in the tissues, including the xylem vessels, which were an important channel for the spread of the pathogen [38,39]. In a survey on grapevine eight months after inoculation, during the emergence of foliar symptoms, plant stress-related responses were modulated in green stems and leaves, especially a downregulation of superoxide dismutase (SOD) and fasciclin-like arabinogalactan protein (fascAGP) and an upregulation of stilbene synthase (STS) genes with an accumulation of phenolics [40]. The ability of the family Botryosphaeriaceae to cause disease may rely on the production of a broad set of effectors, such as cell wall-degrading enzymes, secondary metabolites, and peptidases [41]. Taken together, the two experimental systems showed distinct patterns of metabolic variation following microbial challenge. In the bacterial experiment, the most pronounced differences were observed in carbohydrate metabolism, selected amino acids and secoiridoid-related phenolic compounds, whereas the fungal experiment was characterized primarily by variation in amino acid profiles and more limited changes in major phenolic compounds. Because the two experiments differed in plant material, pathogens and experimental conditions, these findings should be interpreted independently rather than as direct quantitative comparisons.

Because disease severity as well as pathogen re-isolation and quantification of pathogen load were not performed at the time of leaf sampling in parallel with metabolite profiling, the observed metabolic responses should not be interpreted as direct evidence of cultivar resistance or susceptibility. Nevertheless, the results highlight the value of metabolite profiling for characterizing long-term metabolic responses of olive cultivars following bacterial and fungal inoculation. Future studies integrating metabolite analyses with disease severity assessments and additional physiological markers would further improve the understanding of these responses.

## 5. Conclusions

The present study showed that both the bacterial and fungal experiments were associated with changes in primary metabolites and phenolic compounds, whereas significant treatment-related differences in lipid peroxidation were detected only in the bacterial experiment. The bacterial inoculation primarily affected carbohydrate and amino acid metabolism, with particularly pronounced increases in tryptophan concentrations and cultivar-dependent variation in sugars such as sucrose and glucose. In the fungal experiment, amino acids showed greater variability than sugars and organic acids, whereas MDA concentrations did not differ significantly among pathogen treatments.

Phenolic profiling revealed that secoiridoids represented the dominant phenolic class in olive leaves, with oleuropein being the most abundant compound. The coordinated variation observed among oleuropein and related secoiridoids was consistent with differences in the phenolic profiles associated with bacterial treatments. Furthermore, the strong cultivar-dependent differences observed in phenolic composition highlight the importance of genotype in shaping metabolic responses of olive leaves.

Because the bacterial and fungal experiments differed in plant material, pathogens and experimental conditions, their responses should be interpreted independently rather than as directly comparable systems. Nevertheless, both experiments demonstrate that metabolite profiling is a useful approach for characterizing olive responses to microbial challenge and highlight the importance of cultivar-dependent variation in metabolite composition. Future studies integrating metabolite profiling with disease severity assessments and additional physiological markers would provide a more comprehensive understanding of olive–microbe interactions.

## Figures and Tables

**Table 1:** Primary metabolites (sugars, organic acids and free amino acids) detected in olive leaves of the cultivars ‘Istarska bjelica’ (IB) and ‘Rosinjola’ (R) following inoculation with bacterial isolate B45, isolate I7 and the corresponding control. Sugars and organic acids are expressed as g kg^−1^ DW, whereas free amino acids are expressed as mg kg^−1^ DW.

Metabolite	IBB45	IBI7	IBN	RB45	RI7	RN	Cultivar	Isolate	Cultivar × Isolate
‘Istarska Bjelica’				‘Rosinjola’					
**Sugars**									
Glucose	9.63 ± 0.22 a ***	5.81 ± 0.13 c **	6.76 ± 0.11 b	9.32 ± 0.27 a ^ns^	10.27 ± 0.31 a ^ns^	10.04 ± 0.27 a	**<0.001**	**<0.001**	**<0.001**
Fructose	5.80 ± 0.11 a **	5.08 ± 0.33 ab *	3.97 ± 0.35 b	3.92 ± 0.15 a ^ns^	4.60 ± 0.29 a ^ns^	4.31 ± 0.26 a	**0.006**	0.020	**0.002**
Sucrose	86.78 ± 1.39 a *	69.93 ± 2.65 c *	78.87 ± 0.87 b	50.33 ± 0.77 b *	40.53 ± 0.97 c ***	55.04 ± 0.99 a	**<0.001**	**<0.001**	**0.001**
Mannitol	39.66 ± 0.75 a ^ns^	40.05 ± 1.42 a ^ns^	40.16 ± 0.64 a	30.65 ± 0.53 a ^ns^	31.72 ± 0.98 a ^ns^	31.40 ± 0.62 a	**<0.001**	0.673	0.926
**Organic acids**									
Ascorbic acid	1.24 ± 0.17 a ^ns^	1.72 ± 0.34 a ^ns^	1.19 ± 0.22 a	0.77 ± 0.16 b ^ns^	1.53 ± 0.15 a ^ns^	1.25 ± 0.09 ab	0.242	0.022	0.436
Malic acid	2.97 ± 0.87 a ^ns^	3.97 ± 0.06 a ^ns^	4.15 ± 0.19 a	3.28 ± 1.00 a ^ns^	3.16 ± 0.96 a ^ns^	4.59 ± 0.11 a	0.970	0.201	0.602
Quinic acid	18.65 ± 0.81 a ^ns^	21.20 ± 1.16 a ^ns^	23.32 ± 2.90 a	18.80 ± 1.02 a ^ns^	19.67 ± 4.36 a ^ns^	21.99 ± 1.54 a	0.643	0.269	0.926
**Free amino acids**									
Aspartic acid	6.67 ± 0.47 a ^ns^	5.83 ± 0.10 a ^ns^	5.92 ± 1.16 a	7.67 ± 1.05	<LOQ	15.00 ± 2.06	ns	**0.013**	**0.004**
Glutamic acid	9.50 ± 1.11 b *	19.92 ± 2.13 a ^ns^	18.25 ± 2.33 a	9.67 ± 1.01 b ***	10.58 ± 2.21 b ***	25.08 ± 1.40 a	0.600	**<0.001**	**0.001**
Glutamine	<LOQ	8.00	14.47 ± 3.25	9.67 ± 0.69 a ^ns^	11.33 ± 1.02 a ^ns^	11.92 ± 1.81 a	0.858	**0.009**	0.463
Lysine	2.11 ± 0.29 a ^ns^	4.00 ± 0.36 a ^ns^	3.25 ± 0.70 a	3.33 ± 0.24 a ^ns^	4.08 ± 0.34 a ^ns^	3.50 ± 0.55 a	0.215	0.045	0.446
Tryptophan	49.58 ± 3.82 c ***	367.42 ± 21.37 a ***	218.25 ± 17.65 b	131.00 ± 9.99 b ***	96.17 ± 4.62 c ***	228.75 ± 4.86 a	**<0.001**	**<0.001**	**<0.001**

Values are means ± standard error of four biological replicates, each consisting of one olive plant. Different lowercase letters within a cultivar indicate significant differences among the three treatments of that cultivar (Tukey’s HSD, *p* ≤ 0.05); letter “a” denotes the highest mean. Asterisks indicate significant differences from the corresponding control of the same cultivar (Dunnett’s test: * *p* ≤ 0.05, ** *p* ≤ 0.01, *** *p* ≤ 0.001; ns = not significant). “<LOQ” indicates that the analyte was not quantified in any replicate of that treatment, either because it was not detected or because the signal did not meet the acceptance criteria for quantification; limits of quantification for each analyte are given in Supplementary Table S1. Such values were treated as missing and excluded from the analysis. Where a treatment has fewer than two quantifiable replicates, post hoc comparisons could not be performed, and no letter or asterisk is shown. The last three columns give *p*-values of the two-way ANOVA testing the effects of cultivar, isolate and their interaction. DW—dry weight. Treatment abbreviations: IBB45 = ‘Istarska bjelica’ inoculated with isolate B45; IBI7 = ‘Istarska bjelica’ inoculated with isolate I7; IBN = ‘Istarska bjelica’ control; RB45 = ‘Rosinjola’ inoculated with isolate B45; RI7 = ‘Rosinjola’ inoculated with isolate I7; RN = ‘Rosinjola’ control. Statistically significant results (*p* ≤ 0.05) are shown in bold.

**Table 2:** Concentrations of primary metabolites (sugars, organic acids, free amino acids and vitamins) in olive leaves of the cultivars ‘Istarska bjelica’ (IB) and ‘Buža’ (B) after inoculation with *Neofusicoccum parvum*, *Dothiorella iberica* and the corresponding control. Concentrations are expressed as g kg^−1^ DW for sugars and organic acids and mg kg^−1^ DW for free amino acids and vitamins.

Metabolite	IBDOI	IBNP	IBN	BDOI	BNP	BN	Cultivar	Pathogen	Cultivar × Pathogen
	‘Istarska Bjelica’			‘Buža’					
**Sugars**									
Glucose	8.64 ± 1.87 a ^ns^	12.67 ± 3.93 a ^ns^	6.49 ± 1.60 a	26.61 ± 14.17 a ^ns^	10.70 ± 3.45 a ^ns^	12.44 ± 0.76 a	0.169	0.420	0.300
Fructose	2.09 ± 0.22 a ^ns^	1.86 ± 0.67 a ^ns^	1.18 ± 0.47 a	1.71 ± 0.72 a ^ns^	1.95 ± 1.23 a ^ns^	4.70 ± 0.39 a	0.073	0.251	**0.023**
Sucrose	18.85 ± 1.74 a ^ns^	23.77 ± 1.46 a ^ns^	26.46 ± 3.00 a	22.65 ± 0.97 a *	18.70 ± 0.84 b ^ns^	18.58 ± 0.88 b	**0.037**	0.557	**0.007**
**Organic acids**									
Ascorbic acid	0.36 ± 0.05 a ^ns^	0.39 ± 0.09 a ^ns^	0.46 ± 0.11 a	0.35 ± 0.04 a ^ns^	0.33 ± 0.00 a ^ns^	0.27 ± 0.02 a	0.143	0.995	0.388
Citric acid	0.61 ± 0.23 a ^ns^	0.37 ± 0.12 a ^ns^	0.21 ± 0.04 a	0.37 ± 0.20 ab ^ns^	1.06 ± 0.24 a *	0.17 ± 0.03 b	0.258	**0.030**	**0.034**
Malic acid	3.80 ± 0.54 a ^ns^	2.99 ± 0.51 a ^ns^	2.84 ± 0.64 a	2.97 ± 0.57 a ^ns^	3.52 ± 1.33 a ^ns^	2.33 ± 0.23 a	0.649	0.505	0.623
Quinic acid	11.60 ± 0.87 a ^ns^	15.29 ± 1.22 a ^ns^	13.19 ± 1.88 a	9.95 ± 1.17 b **	11.02 ± 0.57 b **	16.47 ± 1.04 a	0.380	**0.011**	**0.017**
Succinic acid	0.10 ± 0.02 a ^ns^	0.10 ± 0.01 a ^ns^	0.08 ± 0.01 a	<LOQ	0.14 ± 0.02	<LOQ	**0.050**	**<0.001**	**0.017**
**Free amino acids and vitamins**									
Aspartic acid	29.25 ± 2.79 a ^ns^	23.50 ± 5.13 a ^ns^	25.50 ± 2.57 a	15.08 ± 2.01	14.83 ± 3.40	<LOQ	**0.004**	0.466	0.629
Alanine	13.56 ± 1.64 a	11.50 ± 1.17 a	6.67	13.33 ± 1.17	20.44 ± 7.95	7.33	0.457	0.379	0.616
Arginine	10.67 ± 4.79 a ^ns^	4.25 ± 1.40 a ^ns^	5.00 ± 0.68 a	11.25 ± 5.82 a ^ns^	6.33 ± 1.25 a ^ns^	2.42 ± 0.32 a	0.992	0.083	0.759
Biotin	<LOQ	6.00 ± 0.67	7.50 ± 1.17	5.00	7.00 ± 0.41	<LOQ	1.000	0.905	0.173
Glutamic acid	49.83 ± 3.59 a ^ns^	43.00 ± 6.84 a ^ns^	52.25 ± 4.79 a	34.92 ± 3.48 a **	43.08 ± 6.90 a ***	8.17 ± 1.55 b	**<0.001**	**0.029**	**0.001**
Glutamine	16.50 ± 4.63 a ^ns^	14.75 ± 3.23 a ^ns^	16.67 ± 5.65 a	12.25 ± 3.73	23.25 ± 4.29	<LOQ	0.778	0.381	0.322
Histidine	2.11 ± 0.44	1.33	1.83 ± 0.22	3.00 ± 0.00	1.78 ± 0.11	<LOQ	**0.026**	0.019	0.526
Isoleucine	18.67 ± 1.21 a *	7.42 ± 2.67 b ^ns^	8.83 ± 2.84 b	9.00 ± 1.59 a ^ns^	10.75 ± 3.57 a ^ns^	9.50 ± 3.44 a	0.403	0.160	0.063
Leucine	11.92 ± 4.71 a ^ns^	9.67 ± 2.03 a ^ns^	9.58 ± 1.80 a	8.67 ± 2.45 a ^ns^	5.00 ± 0.14 a ^ns^	7.50 ± 0.97 a	0.115	0.497	0.873
Lysine	3.33 ± 1.06 a ^ns^	2.83 ± 0.59 a ^ns^	3.58 ± 1.36 a	3.75 ± 1.30	5.42 ± 1.95	1.67	0.455	0.881	0.435
Phenylalanine	29.17 ± 1.60 a ^ns^	29.83 ± 1.08 a ^ns^	29.33 ± 1.19 a	18.33 ± 6.07 ab ^ns^	27.83 ± 1.32 a **	8.17 ± 1.21 b	**<0.001**	**0.006**	**0.009**
Proline	8.33 ± 0.65 a ^ns^	6.67 ± 0.47 a ^ns^	8.67 ± 1.03 a	12.25 ± 2.44 a *	7.33 ± 0.56 ab ^ns^	5.92 ± 0.37 b	0.528	**0.020**	**0.034**
Riboflavin	48.83 ± 7.83 a ^ns^	49.33 ± 5.48 a ^ns^	81.56 ± 33.04 a	68.67 ± 8.67 a ^ns^	59.92 ± 8.52 a ^ns^	51.89 ± 11.21 a	0.912	0.771	0.336
Serine	2.08 ± 0.21 a ^ns^	1.25 ± 0.16 b ^ns^	1.50 ± 0.22 ab	1.56 ± 0.29 a ^ns^	1.00 ± 0.14 a ^ns^	0.83 ± 0.17 a	**0.017**	**0.006**	0.602
Tryptophan	127.33 ± 14.67 a ^ns^	91.75 ± 32.95 a ^ns^	149.17 ± 13.40 a	257.17 ± 77.61	393.00 ± 134.16	<LOQ	**0.009**	0.639	0.407
Valine	8.92 ± 0.86 a ^ns^	7.50 ± 0.69 a ^ns^	8.25 ± 0.86 a	7.75 ± 0.55 a *	7.42 ± 0.60 ab ^ns^	5.00 ± 0.00 b	**0.051**	0.208	0.162

Values are means ± standard error of four biological replicates, each consisting of one olive plant. Different lowercase letters within a cultivar indicate significant differences among the three treatments of that cultivar (Tukey’s HSD, *p* ≤ 0.05); letter “a” denotes the highest mean. Asterisks indicate significant differences from the corresponding control of the same cultivar (Dunnett’s test: * *p* ≤ 0.05, ** *p* ≤ 0.01, *** *p* ≤ 0.001; ns = not significant). “<LOQ” indicates that the analyte was not quantified in any replicate of that treatment, either because it was not detected or because the signal did not meet the acceptance criteria for quantification; limits of quantification for each analyte are given in Supplementary Table S1. Such values were treated as missing and excluded from the analysis. Where a treatment has fewer than two quantifiable replicates, post hoc comparisons could not be performed and no letter or asterisk is shown; where the control of a cultivar was below the LOQ, letters compare the two inoculated treatments only, and no Dunnett comparison was possible. The last three columns give *p*-values of the two-way ANOVA testing the effects of cultivar, pathogen and their interaction. DW—dry weight. Treatment abbreviations: IBDOI = ‘Istarska bjelica’ inoculated with *Dothiorella iberica*; IBNP = ‘Istarska bjelica’ inoculated with *Neofusicoccum parvum*; IBN = ‘Istarska bjelica’ control; BDOI = ‘Buža’ inoculated with *Dothiorella iberica*; BNP = ‘Buža’ inoculated with *Neofusicoccum parvum*; BN = ‘Buža’ control. Statistically significant results (*p* ≤ 0.05) are shown in bold.

## Data Availability

The original contributions presented in this study are included in the article/supplementary material. Further inquiries can be directed to the corresponding author(s).

## Supplementary Materials

The following supporting information can be downloaded at https://www.mdpi.com/article/10.3390/biom16081220/s1. Supplementary Data S1. Raw biological replicate data for sugars, organic acids, free amino acids, phenolic compounds and malondialdehyde (MDA) concentrations from the bacterial and fungal experiments. Supplementary Table S1. Limits of detection (LOD), limits of quantification (LOQ), and coefficients of determination (R^2^) for all analyzed metabolites. Analyte concentrations below the LOQ were reported as “<LOQ” and treated as missing values (NA). Supplementary Table S2: Phenolic compounds detected in olive leaves of the cultivars ‘Istarska bjelica’ and ‘Rosinjola’ following inoculation with bacterial isolates (B45 and I7) and the corresponding controls. Supplementary Table S3: Phenolic compounds detected in olive leaves of the cultivars ‘Istarska bjelica’ and ‘Buža’ following inoculation with the fungal pathogens *Dothiorella iberica* and *Neofusicoccum parvum*, and the corresponding control treatments. Supplementary Table S4: Principal component analysis (PCA) scores for individual samples from the bacterial experiment. Supplementary Table S5: Principal component analysis (PCA) loadings of metabolites from the bacterial experiment. Supplementary Table S6: Principal component analysis (PCA) scores for individual samples from the fungal experiment. Supplementary Table S7: Principal component analysis (PCA) loadings of metabolites from the fungal experiment.
