# Supplementary material for: Long-Term Metabolic Responses of Olive to Bacterial and Fungal Inoculation Differ Between Cultivars
Source: Biomolecules. 2026 Aug 21;16(8):1220. doi: 10.3390/biom16081220 (PMC13511578; doi:10.3390/biom16081220)
Supplement: Supplementary file 1 [file biomolecules-16-01220-s001.zip › Supplementary Table S1.pdf]

Supplementary Table S1. Limits of detection (LOD), limits of quantification (LOQ), and coefficients of determination (R^2) for all analyzed metabolites. Analyte concentrations below the LOQ were reported as "< LOQ" and treated as missing values (NA).

Metabolites	LOQ	LOD	R^2	Quant MRM	Qual MRM	Ratio	Range
**Sugars mg/L**							
Glucose	1.2	0.4	0.9999	179.2000>89.2500	179.2000>59.2000	74.69	37.34 - 112.03
Fructose	2.83	0.93	0.9992	179.2000>89.2500	179.2000>59.2000	60.34	30.17 - 90.51
Sucrose	1.36	0.45	0.9992	341.1000>118.8500	341.1000>89.1500	93.35	46.68 - 140.03
Mannitol	0.18	0.06	0.9997	181.1000>89.1000	181.1000>119.1000	27.46	13.73 - 41.19
**Organic acids mg/L**							
Ascorbic acid	0.041	0.013	0.9993	175.00>115.00	----	----	----
Citric acid	8.628	2.847	0.9995	191.10>110.90	----	----	----
Malic acid	0.351	0.116	0.9992	133.20>115.00	133.20>71.10	48.86	24.43 - 73.29
Quinic acid	1.33	0.44	0.9991	191.15>93.00	191.15>127.10	72.17	36.09 - 108.26
Succinic acid	0.088	0.029	0.9994	117.10>73.00	----	----	----
**Free amino acids ug/L**							
Aspartic acid	14.24	4.7	0.9998	134.05>74.10	134.05>116.05	28.14	14.07 - 42.21
Alanine	52.09	17.19	0.9991	90.05>44.05	----	----	----
Arginine	4.7	1.55	0.9996	175.10>70.10	175.10>130.05	9.25	4.63 - 13.88
Biotin	6.7	2.21	0.9999				
Glutamic acid	4.87	1.61	0.9996	148.05>84.05	----	----	----
Glutamine	11.98	3.48	0.9991	147.10>84.00	147.10>56.05	33.72	16.86 - 50.58
Histidine	4.12	1.36	0.9997	156.10>110.05	156.10>83.05	19.49	9.75 - 29.24
Isoleucine	10.93	3.61	0.9999	132.10>86.10	132.10>44.15	9.81	4.91 - 14.72
Leucine	10.85	3.58	0.9997	132.10>86.10	132.10>30.00	12.77	6.39 - 19.16
Lysine	4.51	1.49	0.9997	147.10>84.05	147.10>130.05	28.52	14.26 - 42.78
Phenylalanine	2.7	0.89	0.9998	166.10>120.10	166.10>103.05	34.08	17.04 - 51.12
Proline	7.22	2.38	0.9992	116.05>70.20	116.05>28.15	4.73	2.37 - 7.10
Riboflavin	6.44	2.13	0.9996	377.15>243.15	377.15>172.15	31.71	15.86 - 47.57
Serine	5.59	1.84	0.9998	106.05>60.15	106.05>42.00	24.73	12.37 - 37.10
Tryptophan	3.97	1.31	0.9998	205.10>188.10	205.10>146.05	45.63	22.82 - 68.45
Valine	4.6	1.52	0.9997	118.10>72.10	118.10>55.05	40.92	20.46 - 61.38
**Polyphenols ug/L**							
2,5-Dihydroxybenzoic acid (Gentisic acid)	7.43	2.45	0.9993	153.10>108.00	153.10>109.05	92.1	46.05 - 138.15
3,4,5-Trihydroxybenzoic acid (gallic acid)	29.78	9.83	0.9996	169.10>125.10	169.10>79.00	10.57	5.28 - 15.85
3,4-Dihydroxybenzoic acid (protocatehuic acid)	2.82	0.93	0.9971	153.00>109.00	153.00>81.00	3.22	1.61 - 4.83
4-hydroxybenzoic acid	15.29	5.05	0.9996	137.00>93.00	----	----	----

Supplementary Table S1. Limits of detection (LOD), limits of quantification (LOQ), and coefficients of determination (R^2) for all analyzed metabolites. Analyte concentrations below the LOQ were reported as "< LOQ" and treated as missing values (NA). (continued)

Apigenin	1.26	0.41	0.9984	270.80>153.00	----	----	----
Apigenin-4-glucoside	7.58	2.5	0.9998	430.90>267.50	----	----	----
Apigenin-7-glucoside	18.32	6.05	0.9992	430.90>267.50	----	----	----
Caffeic acid	3.11	1.03	0.9996	179.10>135.10	----	----	----
Chlorogenic acid	1.15	0.38	0.9705	353.10>191.15	353.10>85.00	5.89	2.95 - 8.83
Dihydroquercetin (taxifolin)	4.62	1.53	0.9992	303.00>285.10	303.00>125.20	27.7	13.85 - 41.56
Ferulic acid	1.46	0.48	0.9994	193.00>134.10	----	----	----
Hydroxytyrosol	0.2	0.07	0.9926	153.00>122.90	----	----	----
Isoferulic acid	28.38	9.37	0.9993	193.10>134.10	193.10>178.05	142.98	71.49 - 214.47
Isorhamnetin	136.23	44.95	0.9991	314.80>300.00	----	----	----
Isorhamnetin-3-O-glucoside	2.16	0.71	0.9999	477.10>314.05	477.10>243.05	52.28	26.14 - 78.43
Isorhamnetin-4'-glucoside	3.09	1.02	0.9996	477.10>314.05	477.10>243.05	52.28	26.14 - 78.43
Luteolin	5.39	1.78	0.9984	286.80>153.00	----	----	----
Luteolin-4-glucoside	22.27	7.35	0.999	447.10>285.00	447.10>133.00	13.13	6.57 - 19.70
Luteolin-4-rutinoside	5.15	1.7	0.9995	593.10>285.05	----	----	----
Luteolin-7-glucoside	6.4	2.11	0.9986	447.10>285.00	447.10>133.00	5.37	2.69 - 8.06
Luteolin-7-rutinoside	5.15	1.7	0.9995	593.10>285.05	----	----	----
Neochlorogenic acid	2.9	0.96	0.9994	353.10>191.10	353.10>179.05	76.35	38.18 - 114.53
Oleacein	11.84	3.91	0.9968	319.10>195.15	319.10>58.95	51.9	25.95 - 77.85
Oleuropein	2.43	0.8	0.9995	539.10>275.10	539.10>307.10	80.03	40.02 - 120.05
Oleuropein aglycone	6	1.98	0.9993	377.10>307.10	377.10>275.10	96.33	48.17 - 144.50
p-Coumaric acid	7.82	2.58	0.9993	163.00>119.00	163.00>93.00	1.44	0.72 - 2.16
Quercetin	7.47	2.47	0.9987	301.00>179.00	301.00>151.00	86.73	43.36 - 130.09
Quercetin-3,4'-diglucoside	41.18	13.59	0.9995	624.80>463.10	----	----	----
Quercetin-3-glucoside (Isorquercitroside)	7.26	2.4	0.9995	462.90>271.10	462.90>271.10	100	50.00 - 150.00
Quercetin-3-rhamnoside (quercitrin)	1.6	0.5	0.9999	446.90>301.00	----	----	----
Quercetin-3-rutinoside (rutin)	2.49	0.82	0.9992	608.90>300.10	----	----	----
Quercetin-4'-glucoside (spiraeoside)	5.47	1.8	0.9998	462.90>301.00	462.90>271.10	0.36	0.18 - 0.54
Vanillic acid	2.53	0.83	0.9983	167.00>152.20	167.00>107.50	8.14	4.07 - 12.21
Vanillic acid-4-glucoside	0.61	0.2	0.9972	329.10>167.30	329.10>151.50	21.98	10.99 - 32.96
Vanillin	2.53	0.83	0.9985	151.10>135.90	----	----	----
Verbascoside	20.91	6.9	0.9999	622.90>161.10	622.90>461.10	21.88	10.94 - 32.82

Analyte concentrations below the limit of quantification (< LOQ) were treated as missing values (NA), were not replaced by zeros or imputed values, and were excluded from inferential statistical analyses as described in Materials and Methods (Section 2.7).
