# Supplementary material for: Long-Term Metabolic Responses of Olive to Bacterial and Fungal Inoculation Differ Between Cultivars
Source: Biomolecules. 2026 Aug 21;16(8):1220. doi: 10.3390/biom16081220 (PMC13511578; doi:10.3390/biom16081220)
Supplement: Supplementary file 1 [file biomolecules-16-01220-s001.zip › Supplementary Table S2.pdf]

Supplementary Table S2. Phenolic compounds detected in olive leaves of the cultivars 'Istarska bjelica' and 'Rosinjola' following inoculation with bacterial isolates (B45 and I7) and the corresponding controls.

Phenolic compound	IBB45	IBI7	IBN (control)	RB45	RI7	RN (control)	Cultivar	Isolate	Cultivar × Isolate
	'Istarska bjelica'			'Rosinjola'					
Luteolin	7.89 ± 0.15 a ns	6.33 ± 0.26 a ns	8.30 ± 4.40 a	5.78 ± 0.06 a ns	27.36 ± 12.31 a ns	3.70 ± 0.14 a	0.288	0.105	**0.052**
Apigenin	3.64 ± 0.07 a ns	3.20 ± 0.11 a ns	5.04 ± 2.62 a	5.24 ± 2.59 a ns	13.94 ± 6.45 a ns	2.47 ± 0.18 a	0.205	0.255	0.109
Vanillic acid-4-O-glucoside	13.66 ± 0.74 a ns	13.49 ± 1.27 a ns	15.05 ± 0.26 b	24.70 ± 0.99 a ns	19.62 ± 0.75 b ***	27.15 ± 0.44 a	**<0.001**	**<0.001**	**0.004**
Hydroxytyrosol	45.43 ± 0.96 ab ns	42.38 ± 1.56 b *	49.16 ± 1.46 a	56.50 ± 2.02 ab ns	60.95 ± 0.99 b **	53.26 ± 1.13 a	**<0.001**	0.882	**<0.001**
Esculin	3.50 ± 0.19 a ns	3.61 ± 0.33 a ns	2.95 ± 0.18 a	3.79 ± 0.15 a ns	3.47 ± 0.26 a ns	3.36 ± 0.36 a	0.383	0.158	0.536
4-hydroxybenzoic acid	13.03 ± 0.88 ab ns	10.17 ± 0.68 b *	13.35 ± 0.63 a	5.31 ± 0.40 a ns	5.16 ± 0.46 a ns	4.68 ± 0.18 a	**<0.001**	**0.036**	**0.016**
Chlorogenic acid	8.51 ± 0.85 a ns	8.13 ± 0.32 a ns	7.34 ± 0.43 a	27.61 ± 1.20 a ns	28.18 ± 0.89 a ns	24.36 ± 1.56 a	**<0.001**	0.049	0.305
Caffeic acid	11.28 ± 0.58 a ns	22.02 ± 0.48 b ***	13.26 ± 0.63 a	29.45 ± 1.21 b ***	27.02 ± 1.26 b ***	17.30 ± 1.23 a	**<0.001**	**<0.001**	**<0.001**
Secologanoside	1692.98 ± 49.17 a ns	964.95 ± 28.46 b ***	1699.54 ± 47.70 a	2737.97 ± 111.95 b **	2238.40 ± 113.73 c ***	3378.17 ± 69.86 a	**<0.001**	**<0.001**	**0.002**
11-methyloleoside	708.51 ± 72.52 a ns	545.60 ± 39.29 a ns	579.17 ± 20.89 a	1242.41 ± 94.74 a ns	1163.55 ± 48.68 a ns	1168.79 ± 96.79 a	**<0.001**	0.192	0.824
4-Hydroxycinnamic acid (p-coumaric)	66.15 ± 2.46 b *	125.55 ± 5.08 c ***	49.37 ± 3.93 a	92.53 ± 6.57 b ***	83.63 ± 4.85 b ***	27.98 ± 2.03 a	**0.003**	**<0.001**	**<0.001**
Dihydroquercetin (taxifolin)	28.43 ± 1.98 b **	20.65 ± 0.83 a ns	19.63 ± 1.11 a	26.80 ± 1.66 a ns	27.34 ± 1.30 a ns	28.85 ± 0.26 a	**<0.001**	**0.022**	**0.002**
Quercetin-3,4'-O-diglucoside	6.85 ± 0.41 a ns	8.91 ± 0.50 b **	6.09 ± 0.51 a	11.40 ± 1.12 b *	7.70 ± 1.18 ab ns	5.81 ± 1.34 a	0.195	**0.008**	**0.013**
Ferulic acid	15.35 ± 0.23 a ns	27.12 ± 3.30 b *	19.17 ± 0.35 a	49.02 ± 0.87 b ***	48.46 ± 0.88 b ***	24.12 ± 0.50 a	**<0.001**	**<0.001**	**<0.001**
Luteolin-3,7-O-glucoside	105.52 ± 4.87 a ns	210.71 ± 12.52 b *	144.79 ± 22.31 a	80.69 ± 4.76 b **	72.19 ± 2.98 b *	52.78 ± 3.72 a	**<0.001**	**0.001**	**<0.001**

Apigenin-8-C-glucoside (Vitexin)	-	-	-	7.06 ± 1.51 a ns	6.73 ± 0.87 a ns	4.33 ± 0.23 a	**<0.001**	1.000	0.171
Verbascoside	4572.57 ± 255.83 a ns	4854.56 ± 292.47 a ns	4105.35 ± 183.44 a	1440.18 ± 65.40 b **	1601.06 ± 37.09 ab ns	1778.30 ± 79.78 a	**<0.001**	0.279	**0.040**
Naringenin-7-O-rutinoside (Narirutin)	3.70 ± 0.24 a ns	3.98 ± 0.46 a ns	3.70 ± 0.21 a	7.54 ± 0.55 b ***	5.76 ± 0.19 c *	4.27 ± 0.18 a	**<0.001**	**0.001**	**<0.001**
Apigenin-6-C-glucoside (Isovitexin)	-	-	-	3.18 ± 0.27 b *	2.79 ± 0.18 ab ns	2.31 ± 0.08 a	**<0.001**	1.000	**0.032**
Luteolin-7-O-glucoside	859.17 ± 24.78 a ns	779.69 ± 42.44 a ns	699.68 ± 78.39 a	1719.11 ± 116.20 b **	1533.19 ± 171.13 b *	1011.30 ± 49.30 a	**<0.001**	**0.001**	**0.023**
Oleacein	871.02 ± 69.30 a ***	409.94 ± 26.96 b ns	319.82 ± 18.77 b	645.25 ± 53.35 a ns	1455.07 ± 265.35 b ***	162.16 ± 14.78 a	**0.030**	**<0.001**	**<0.001**
Luteolin-7-O-neohesperidoside	122.68 ± 5.64 a **	96.59 ± 5.13 ab ns	86.04 ± 8.62 b	120.78 ± 16.86 a ns	111.62 ± 15.35 a ns	140.40 ± 8.01 a	**0.021**	0.297	0.052
Hesperetin-7-O-rutinoside (Hesperidin)	4.42 ± 0.39 a **	4.88 ± 0.09 a ***	2.98 ± 0.11 b	3.03 ± 0.20 a ns	2.74 ± 0.10 a ns	2.58 ± 0.22 a	**<0.001**	**<0.001**	**0.003**
Quercetin-3-O-rutinoside (rutin)	254.52 ± 10.80 b ***	268.24 ± 10.83 b ***	162.94 ± 14.20 a	157.08 ± 7.34 a ns	145.84 ± 5.94 a ns	152.96 ± 10.97 a	**<0.001**	**<0.001**	**<0.001**
Quercetin-3-O-glucoside (Isorquercitroside)	71.67 ± 3.80 b ***	54.19 ± 5.20 c *	34.18 ± 2.64 a	56.02 ± 3.89 a ns	58.90 ± 1.18 a .	46.84 ± 4.12 a	0.853	**<0.001**	**0.004**
Apigenin-7-O-rutinoside (Isorhoifolin)	333.51 ± 9.50 a ns	352.79 ± 18.07 a ns	355.99 ± 32.06 a	335.09 ± 14.06 ab ns	372.76 ± 27.76 a *	297.23 ± 9.30 b	0.466	0.203	0.160
Oleuroside	1950.71 ± 59.10 b **	2394.62 ± 77.28 a ns	2439.79 ± 99.22 a	2766.80 ± 38.79 b *	2302.42 ± 43.64 a ns	2409.25 ± 119.35 a	**0.002**	0.585	**<0.001**
Apigenin-7-O-neohesperidoside (Rhoifolin)	7.22 ± 0.31 a ns	6.01 ± 0.46 a ns	7.85 ± 0.89 a	4.39 ± 1.30 a ns	7.91 ± 3.01 a ns	2.61 ± 0.47 a	0.093	0.477	0.061
Oleuropein	27898.75 ± 442.34 b **	30644.44 ± 547.90 a ns	31608.72 ± 592.60 a	38275.94 ± 541.40 b ***	32942.76 ± 546.12 a ns	33048.15 ± 447.20 a	**<0.001**	0.070	**<0.001**

Quercetin-4'-O-glucoside (spiraeoside)	13.24 ± 0.76 b ***	4.25 ± 0.54 a ns	1.98 ± 0.69 a	8.22 ± 3.26 a ns	12.56 ± 4.99 a ns	10.26 ± 0.61 a	0.074	0.209	**0.022**
Quercetin-3-O-rhamnoside (quercitrin)	139.21 ± 3.33 b ***	104.72 ± 4.28 a ns	81.19 ± 11.21 a	46.68 ± 1.51 b **	44.39 ± 3.27 b **	58.14 ± 1.01 a	**<0.001**	**0.001**	**<0.001**
Luteolin-4'-O-glucoside	250.51 ± 19.34 a ns	201.68 ± 3.22 a ns	198.83 ± 58.79 a	403.55 ± 104.38 a ns	388.35 ± 101.21 a ns	154.74 ± 5.02 a	0.078	0.075	0.184
Kaempferol-3-O-glucoside	3.10 ± 0.15 a ns	2.34 ± 0.14 a ns	2.28 ± 0.45 a	4.97 ± 1.34 a ns	5.92 ± 1.80 a ns	2.14 ± 0.12 a	0.033	0.098	0.168
Isorhamnetin-3-O-glucoside	6.63 ± 0.80 a ns	6.81 ± 0.64 a ns	5.45 ± 0.60 a	14.92 ± 1.15 ab *	15.30 ± 0.71 b *	10.59 ± 1.42 a	**<0.001**	**0.008**	0.159
Oleocanthal	17.46 ± 1.49 b ***	7.60 ± 0.40 a ns	7.42 ± 0.18 a	5.80 ± 0.45	7.37 ± 0.54	-	ns	**<0.001**	**<0.001**
Isorhamnetin-3-O-rutinoside	6.51 ± 0.30 a ns	13.62 ± 0.45 b ***	6.65 ± 0.59 a	23.12 ± 1.65 b *	19.24 ± 0.96 ab ns	16.27 ± 1.61 a	**<0.001**	**0.001**	**<0.001**
Oleuropein aglycone	338.14 ± 13.98 b *	313.77 ± 8.33 ab ns	280.24 ± 11.97 a	1033.25 ± 25.82 b ***	773.67 ± 28.74 c ***	298.12 ± 6.22 a	**<0.001**	**<0.001**	**<0.001**
Quercetin	-	-	-	56.49 ± 33.16 a ns	127.37 ± 65.47 a ns	15.63 ± 2.34 a	**0.024**	1.000	0.223
Naringenin	2.59 ± 0.09 a ns	2.62 ± 0.06 a ns	2.54 ± 0.26 a	3.63 ± 0.38 a ns	4.59 ± 0.85 a ns	3.03 ± 0.25 a	**0.003**	0.163	0.216
Isorhamnetin	1.27 ± 0.03	-	-	20.42 ± 12.61 a ns	48.64 ± 25.93 a ns	7.27 ± 0.43 a	0.172	1.000	0.159
Total analyzed phenolics	40459.31 ± 493.15 b **	42540.15 ± 596.40 a ns	43045.84 ± 375.65 a	51561.71 ± 764.88 b ***	45823.91 ± 869.87 a ns	44461.63 ± 266.55 a	**<0.001**	**0.003**	**<0.001**

Different lowercase letters within rows indicate significant differences among treatments according to Tukey's HSD test ($p \leq 0.05$). Asterisks indicate significant differences compared with the corresponding control according to Dunnett's test (*$p \leq 0.05$, **$p \leq 0.01$, ***$p \leq 0.001$; ns – not significant). Values represent mean ± standard error (SE) of biological replicates. The last three columns present p-values from two-way ANOVA, testing the effects of cultivar, isolate and their interaction. Treatment abbreviations: IBB45 = 'Istarska bjelica' inoculated with bacterial isolate B45; IBI7 = 'Istarska bjelica' inoculated with bacterial isolate I7; IBN = 'Istarska bjelica' negative control; RB45 = 'Rosinjola' inoculated with bacterial isolate B45; RI7 = 'Rosinjola' inoculated with bacterial isolate I7; RN = 'Rosinjola' negative control.
