# Supplementary material for: Long-Term Metabolic Responses of Olive to Bacterial and Fungal Inoculation Differ Between Cultivars
Source: Biomolecules. 2026 Aug 21;16(8):1220. doi: 10.3390/biom16081220 (PMC13511578; doi:10.3390/biom16081220)
Supplement: Supplementary file 1 [file biomolecules-16-01220-s001.zip › Supplementary Table S3.pdf]

Supplementary Table S3. Phenolic compounds detected in olive leaves of the cultivars 'Istarska bjelica' and 'Buža' following inoculation with the fungal pathogens *Dothiorella iberica* and *Neofusicoccum parvum*, and the corresponding control treatments.

Phenolic compound	IBDOI	IBNP	IBN (control)	BDOI	BNP	BN (control)	Cultivar	Pathogen	Cultivar × Pathogen
	'Istarska bjelica'			'Buža'					
Coniferyl alcohol	8.51 ± 0.33 a ns	7.44 ± 0.93 a ns	7.46 ± 0.63 a	4.57 ± 1.19 a ns	6.46 ± 1.16 a ns	6.33 ± 0.60 a	**0.010**	0.878	0.185
Baicalein-7-O-glucuronide (Baicalin)	0.09 ± 0.03 b *	0.05 ± 0.01 b **	0.18 ± 0.02 a	0.11 ± 0.02 a *	0.09 ± 0.03 a *	0.27 ± 0.05 b	**0.031**	**<0.001**	0.348
Luteolin	9.53 ± 1.11 a ns	9.68 ± 2.95 a ns	7.51 ± 2.02 a	5.68 ± 0.76 a ns	13.34 ± 4.21 a ns	8.16 ± 1.03 a	0.938	0.206	0.301
Apigenin	1.88 ± 0.17 a ns	2.54 ± 0.73 a ns	1.97 ± 0.36 a	1.55 ± 0.19 a ns	3.18 ± 1.31 a ns	3.27 ± 0.21 a	0.321	0.199	0.455
Cinnamic acid methylester (methyl cinnamat)	2.05 ± 0.20 a ns	2.96 ± 0.81 a ns	3.24 ± 0.64 a	2.10 ± 0.22 b **	1.77 ± 0.45 b **	4.12 ± 0.34 a	0.833	**0.010**	0.136
3,4-Dihydroxybenzoic acid (protocatehuic acid)	2.39 ± 0.17 a ns	2.83 ± 0.31 a ns	2.70 ± 0.17 a	2.94 ± 0.13 a ns	3.59 ± 0.33 a ns	3.37 ± 0.26 a	**0.003**	0.096	0.911
Vanillic acid-4-O-glucoside	34.01 ± 1.19 b **	26.11 ± 2.95 ab ns	22.00 ± 2.35 a	15.21 ± 4.37 a ns	14.32 ± 1.75 a ns	8.18 ± 0.64 a	**<0.001**	**0.005**	0.380
Hydroxytyrosol	42.68 ± 5.92 b **	17.15 ± 2.67 a ns	19.86 ± 2.83 a	18.73 ± 1.74 a ns	41.72 ± 8.34 b ns	30.27 ± 5.31 ab	0.382	0.512	**<0.001**
Neochlorogenic acid	2.84 ± 0.17 b *	1.86 ± 0.10 a ns	2.03 ± 0.21 a	10.71 ± 5.34 a ns	6.95 ± 1.63 a ns	6.35 ± 0.37 a	**0.006**	0.471	0.722
Esculin	8.07 ± 1.14 a ns	5.02 ± 1.07 a ns	4.97 ± 0.41 a	4.90 ± 1.47 ab ns	10.00 ± 1.96 b *	3.96 ± 0.32 a	0.788	0.060	**0.009**
4-hydroxybenzoic acid	4.32 ± 0.36 a ns	7.17 ± 1.13 b ns	4.48 ± 0.27 ab	5.53 ± 1.10 b **	5.34 ± 2.23 b **	17.27 ± 2.44 a	**0.004**	**0.002**	**0.000**
Chlorogenic acid	14.74 ± 1.27 a ns	17.23 ± 1.25 a ns	14.74 ± 0.84 a	47.42 ± 25.11 b **	50.84 ± 10.71 b **	142.84 ± 5.01 a	**<0.001**	**0.001**	**0.001**
Cryptochlorogenic acid	2.94 ± 0.23 a ns	2.51 ± 0.17 a ns	2.46 ± 0.46 a	11.00 ± 5.60 a ns	6.29 ± 1.37 a ns	10.38 ± 0.39 a	**0.003**	0.534	0.600

Esculetin	2.45 ± 0.17 a ns	2.95 ± 0.65 a ns	2.75 ± 0.49 a	3.11 ± 0.71 a ns	3.67 ± 0.31 a ns	3.56 ± 0.20 a	0.075	0.527	0.987
Vanillic acid	3.44 ± 0.52 a ns	3.12 ± 0.58 a ns	2.05 ± 0.28 a	2.37 ± 0.47 a ns	2.73 ± 0.75 a ns	3.46 ± 0.36 a	0.970	0.939	0.071
Caffeic acid	6.68 ± 1.41 a ns	4.78 ± 0.84 a ns	5.00 ± 1.13 a	7.00 ± 2.49 a ns	8.50 ± 1.55 a ns	4.64 ± 4.10 a	0.508	0.611	0.622
Secologanoside	1149.86 ± 108.56 a ns	1279.60 ± 346.69 a ns	1256.63 ± 69.71 a	2017.01 ± 567.26 a ns	1433.03 ± 218.31 a ns	2453.81 ± 195.91 a	**0.008**	0.278	0.236
11-methyloleoside	628.95 ± 146.34 a ns	686.17 ± 61.97 a ns	831.55 ± 36.55 a	775.01 ± 127.69 a ns	672.41 ± 158.85 a ns	453.28 ± 30.24 a	0.362	0.856	0.068
Vanillin	1.02 ± 0.06 b *	0.87 ± 0.05 ab ns	0.77 ± 0.05 a	0.83 ± 0.09 a ns	0.82 ± 0.14 a ns	1.01 ± 0.14 a	0.991	0.751	0.107
Isoscopoletin	0.38 ± 0.10 a ns	0.71 ± 0.13 a ns	0.66 ± 0.09 a	0.71 ± 0.13 a ns	0.40 ± 0.11 a ns	0.67 ± 0.09 a	**0.006**	0.133	0.195
Scopoletin	0.16 ± 0.03 a ns	0.29 ± 0.06 a ns	0.20 ± 0.03 a	0.29 ± 0.88 a ns	0.18 ± 0.04 a ns	0.13 ± 0.01 a	0.056	0.047	0.477
4-Hydroxycinnamic acid (p-coumaric)	3.71 ± 0.49 a ns	4.68 ± 0.88 a ns	3.82 ± 1.42 a	5.06 ± 0.78 b ***	6.29 ± 0.40 b ***	0.53 ± 0.06 a	0.867	**0.002**	**0.010**
Dihydroquercetin (taxifolin)	4.84 ± 0.47 a ns	7.06 ± 1.14 a ns	7.66 ± 1.22 a	7.07 ± 1.71 a ns	15.28 ± 4.35 a ns	5.31 ± 0.42 a	0.123	**0.038**	**0.058**
Quercetin-3,4'-O-diglucoside	9.93 ± 0.98 a ns	5.86 ± 0.54 b ns	9.10 ± 0.64 a	6.40 ± 0.46 a ns	9.53 ± 1.96 a ns	5.49 ± 1.75 a	0.256	0.775	**0.010**
Ferulic acid	7.43 ± 1.38 a ns	4.84 ± 0.52 a ns	6.10 ± 0.83 a	13.19 ± 4.32 a ns	9.15 ± 1.64 a ns	16.55 ± 0.83 a	**0.001**	0.113	0.313
2-Hydroxybenzoic acid (Salicyclic acid)	2.44 ± 0.49 a ns	2.39 ± 0.77 a ns	2.00 ± 0.26 a	1.73 ± 0.50 a ns	1.52 ± 0.16 a ns	0.83 ± 0.59 a	**0.038**	0.392	0.899
Luteolin-3,7-O-glucoside	516.21 ± 19.17 b ***	335.83 ± 23.36 a ns	311.86 ± 23.09 a	330.30 ± 82.01 b *	314.49 ± 32.01 ab *	122.01 ± 5.66 a	**0.001**	**<0.001**	0.075
Apigenin-8-C-glucoside (Vitexin)	0.02 ± 0.00 a ns	0.02 ± 0.01 a ns	0.02 ± 0.01 a	0.03 ± 0.01 a ns	0.02 ± 0.00 a ns	0.01 ± 0.00 a	0.852	0.627	0.171
Verbascoside	4694.71 ± 249.93 a ns	4012.70 ± 228.92 a ns	4044.62 ± 191.73 a	2386.23 ± 603.76 a	4240.85 ± 414.70 b ns	3919.89 ± 137.12 ab	**0.017**	0.232	**0.003**
Isoferulic acid	3.77 ± 0.57 b *	2.40 ± 0.47 ab ns	1.70 ± 0.38 a	3.78 ± 1.14 b **	4.25 ± 0.42 b **	9.40 ± 0.76 a	**<0.001**	**0.010**	**<0.001**

Apigenin-6-C-glucoside (Isovitexin)	0.53 ± 0.03 a ns	0.64 ± 0.10 a ns	0.70 ± 0.15 a	0.42 ± 0.01 a ns	0.45 ± 0.02 a ns	0.36 ± 0.14 a	**0.013**	0.769	0.476
Naringenin-7-O-glucoside	9.15 ± 0.53 a ns	7.89 ± 0.56 a ns	8.83 ± 1.12 a	6.22 ± 1.33 a ns	6.94 ± 0.15 a ns	5.42 ± 0.41 a	**0.002**	0.784	0.287
Luteolin-7-O-glucoside	1997.36 ± 54.77 a ns	1730.41 ± 60.78 a ns	1819.12 ± 140.47 a	1635.98 ± 47.04 b **	1811.32 ± 47.68 b ***	1338.55 ± 52.61 a	**0.001**	0.012	**0.004**
Luteolin-7-O-neohesperidoside	55.30 ± 15.57 a ns	89.75 ± 6.05 a ns	73.35 ± 9.89 a	55.42 ± 9.05 a ns	42.57 ± 8.36 a ns	29.91 ± 2.42 a	**0.001**	0.303	**0.040**
Naringenin-4-O-glucoside	0.32 ± 0.01 a ns	0.27 ± 0.03 a ns	0.30 ± 0.05 a	0.12 ± 0.02 a ns	0.17 ± 0.02 a ns	0.95 ± 0.49 a	0.478	0.096	**0.100**
Apigenin-7-O-rutinoside (Isorhoifolin)	273.85 ± 13.61 a ns	185.79 ± 11.60 b ns	225.17 ± 27.24 ab	222.41 ± 10.37 b ns	174.61 ± 3.43 c ns	109.64 ± 3.63 a	**<0.001**	**<0.001**	**0.006**
Quercetin-3-O-arabinoside (guajaverin)	0.31 ± 0.05 a ns	0.36 ± 0.04 a ns	0.49 ± 0.08 a	0.40 ± 0.06 b *	0.33 ± 0.04 b **	0.63 ± 0.06 a	0.165	**0.001**	0.295
Oleuroside	5571.92 ± 277.45 a ns	6165.25 ± 524.23 a ns	6035.06 ± 205.89 a	5747.49 ± 264.53 a ns	5955.63 ± 355.06 a ns	6101.06 ± 139.62 a	0.968	0.363	0.826
Oleuropein	30281.66 ± 1853.11 a ns	32274.15 ± 1344.17 a ns	32639.60 ± 420.15 a	29727.53 ± 407.99 a ns	29766.79 ± 1112.36 a ns	30625.40 ± 877.00 a	0.082	0.364	0.671
Quercetin-4'-O-glucoside (spiraeoside)	1.91 ± 0.28 a ns	1.13 ± 0.13 a ns	1.45 ± 0.26 a	2.32 ± 0.39 b **	2.46 ± 0.48 b **	0.44 ± 0.09 a	0.332	**0.004**	**0.004**
Quercetin-3-O-rhamnoside (quercitrin)	176.38 ± 48.60 b *	229.87 ± 19.53 ab ns	301.38 ± 13.32 a	81.25 ± 11.06 a ns	244.08 ± 29.85 b ***	52.97 ± 3.95 a	**<0.001**	**0.002**	**<0.001**
Luteolin-4'-O-glucoside	747.94 ± 30.09 a .	560.44 ± 52.00 b ns	598.50 ± 54.88 ab	538.37 ± 43.45 b **	570.61 ± 20.81 b **	368.54 ± 15.55 a	**<0.001**	**0.003**	**0.011**
Kaempferol-3-O-glucoside	10.80 ± 0.86 b *	7.34 ± 0.84 a ns	7.25 ± 0.82 a	7.02 ± 0.71 b *	8.21 ± 0.33 b **	4.85 ± 0.24 a	**0.005**	**0.002**	**0.009**
Kaempferol-3-O-rutinoside (camelliaside A)	5.95 ± 1.83 a ns	7.17 ± 0.91 a ns	7.33 ± 0.51 a	3.35 ± 0.27 b **	4.31 ± 0.35 b ***	1.59 ± 0.24 a	**<0.001**	0.317	0.172

Isorhamnetin-3-O-glucoside	2.57 ± 0.38 b *	3.55 ± 0.34 ab ns	4.37 ± 0.47 a	2.15 ± 0.35 a **	5.61 ± 0.86 b ***	2.54 ± 0.22 a	0.876	**0.001**	**0.003**
Isorhamnetin-3-O-rutinoside	9.08 ± 3.24 a ns	12.54 ± 1.85 a ns	15.45 ± 1.32 a	10.68 ± 4.78 ab ns	26.36 ± 5.63 b *	5.50 ± 0.56 a	0.522	**0.020**	**0.010**
trans-Cinnamic acid	2.01 ± 0.24 a ns	2.56 ± 0.30 a ns	2.96 ± 0.29 a	1.45 ± 0.10 a ns	1.40 ± 0.19 a *	2.04 ± 0.18 a	**<0.001**	**0.011**	0.439
Kaempferol-3-O-rhamnoside	0.62 ± 0.31 a ns	0.98 ± 0.09 a ns	1.21 ± 0.14 a	0.23 ± 0.02 a ns	0.60 ± 0.14 b **	0.15 ± 0.00 a	**<0.001**	0.078	0.069
Isorhamnetin-3-O-rhamnoside	0.30 ± 0.05 a *	0.31 ± 0.03 a ns	0.39 ± 0.02 a	0.19 ± 0.01 a ns	0.48 ± 0.08 b *	0.39 ± 0.02 ab	0.779	**0.022**	**0.005**
Oleuropein aglycone	275.97 ± 31.44 a ns	1022.16 ± 299.91 a ns	784.04 ± 212.77 a	957.93 ± 333.76 a ns	1255.11 ± 505.09 a ns	853.57 ± 99.20 a	0.186	0.225	0.565
Quercetin	5.85 ± 0.24 ab ns	4.09 ± 0.45 b *	8.07 ± 1.35 a	7.90 ± 1.43 a ns	35.47 ± 7.81 b **	4.65 ± 0.96 a	**0.002**	**0.001**	**<0.001**
Naringenin	2.29 ± 0.10 b **	1.32 ± 0.22 a ns	1.24 ± 0.18 a	1.93 ± 0.91 a ns	2.67 ± 0.86 a ns	0.83 ± 0.06 a	0.657	0.113	0.197
Kaempferol	3.20 ± 0.29 a ns	2.85 ± 0.65 a ns	3.14 ± 0.72 a	1.96 ± 0.18 a ns	4.06 ± 0.85 a ns	2.66 ± 0.22 a	0.717	0.273	0.115
Isorhamnetin	0.89 ± 0.13 b **	1.14 ± 0.13 b *	1.70 ± 0.12 a	2.06 ± 0.18 a ns	5.17 ± 0.99 b **	1.25 ± 0.27 a	**<0.001**	**0.001**	**<0.001**
Diosmetin	8.35 ± 1.89 a ns	6.43 ± 2.27 a ns	5.62 ± 0.88 a	9.44 ± 1.81 a ns	13.03 ± 4.98 a ns	12.29 ± 1.72 a	0.037	0.938	0.483
Total analyzed phenolics	46614.52 ± 1969.80 a ns	48774.50 ± 2325.19 a ns	49126.02 ± 336.22 a	44713.31 ± 437.99 a ns	46825.43 ± 1784.81 a ns	46770.50 ± 971.18 a	0.111	0.265	0.986

Different lowercase letters within rows indicate significant differences among treatments according to Tukey's HSD test ($p \leq 0.05$). Asterisks indicate significant differences compared with the corresponding control according to Dunnett's test (*$p \leq 0.05$, **$p \leq 0.01$, ***$p \leq 0.001$; ns – not significant). Values represent mean ± standard error (SE) of biological replicates. The last three columns present p-values from two-way ANOVA, testing the effects of cultivar, pathogen treatment and their interaction. Treatment abbreviations: IBNP = 'Istarska bjelica' inoculated with *Neofusicoccum parvum*; IBDOI = 'Istarska bjelica' inoculated with *Dothiorella iberica*; IBN = 'Istarska bjelica' negative control; BNP = 'Buža' inoculated with *Neofusicoccum parvum*; BDOI = 'Buža' inoculated with *Dothiorella iberica*; BN = 'Buža' negative control.
