# Supplementary material for: Long-Term Metabolic Responses of Olive to Bacterial and Fungal Inoculation Differ Between Cultivars
Source: Biomolecules. 2026 Aug 21;16(8):1220. doi: 10.3390/biom16081220 (PMC13511578; doi:10.3390/biom16081220)
Supplement: Supplementary file 1 [file biomolecules-16-01220-s001.zip › Supplementary Table S4.pdf]

Supplementary Table S4. Principal component analysis (PCA) scores for individual samples from the bacterial experiment.

Sample	PC1	PC2	PC3	PC4	PC5	PC6	PC7	PC8	PC9	PC10	PC11	PC12	PC13
IBB451	-0.042	-2.710	-1.029	-2.253	2.822	1.593	0.875	-1.368	-0.472	0.018	-0.071	-0.156	-0.003
IBB452	0.449	-2.881	0.065	-1.594	-0.490	-0.015	-0.364	0.635	0.269	0.340	0.499	0.089	0.125
IBB453	-0.278	-3.336	0.602	0.939	-2.993	0.624	-0.532	-0.646	0.253	0.051	0.126	-0.049	-0.072
IBB454	-0.289	-2.437	0.723	0.363	-0.499	1.023	-1.231	0.762	0.081	-0.147	-0.461	-0.409	0.117
IBI71	-2.412	1.054	-0.200	2.748	1.197	0.591	0.082	-0.135	0.321	-0.207	0.155	-0.335	0.190
IBI72	-2.741	0.607	-0.284	0.136	-0.527	-0.258	0.644	0.555	-1.583	0.254	0.427	-0.171	-0.343
IBI73	-1.877	-0.055	-0.340	0.722	0.593	-0.460	0.436	0.343	0.606	-0.695	0.468	0.010	-0.040
IBI74	-3.467	0.066	-1.250	0.842	-0.514	0.810	1.168	0.808	-0.621	0.263	-0.222	0.242	0.143
IBN1	-0.987	0.337	-1.341	-1.028	-0.621	-1.830	-0.402	-0.446	-0.307	-0.211	-0.405	-0.095	-0.180
IBN2	-3.348	1.208	3.651	-1.535	-0.206	-0.109	-0.244	-0.894	-0.310	-0.285	0.136	0.250	0.199
IBN3	-1.712	-0.360	-1.718	0.163	0.186	-1.278	-0.199	-0.300	1.020	0.452	-0.726	0.158	0.003
IBN4	-1.358	-1.024	-0.087	0.259	1.229	-1.175	-1.271	-0.308	0.586	-0.117	-0.041	0.316	-0.181
RB451	2.761	-0.281	-0.271	0.646	-1.054	-1.400	0.817	-1.050	-1.385	-0.285	-0.265	-0.099	0.109
RB452	1.308	-0.015	0.517	0.054	0.731	-0.468	0.329	1.126	-0.001	0.235	-0.081	-0.005	0.393
RB453	1.578	0.055	0.363	-0.421	0.702	-1.633	0.495	0.774	0.852	-0.337	0.171	-0.243	0.136
RB454	1.853	0.209	0.208	-0.672	0.321	-1.356	-0.401	0.601	-0.914	0.144	0.405	-0.024	0.038
RI71	2.186	-0.468	1.090	1.612	1.609	0.702	-0.957	0.711	-0.950	0.157	-0.146	0.385	-0.160
RI72	1.346	1.238	1.285	0.409	0.470	0.397	0.120	0.233	0.190	0.550	-0.460	-0.176	-0.391
RI73	2.703	-0.437	0.077	2.291	-0.103	0.149	1.040	-1.366	0.500	-0.005	0.329	0.237	0.040
RI74	0.948	0.529	1.643	-1.115	-0.709	0.694	1.806	0.686	1.019	-0.422	-0.367	0.051	-0.340
RN1	0.215	3.240	0.768	-0.187	0.077	0.276	-1.066	-1.122	0.389	0.571	0.203	-0.278	0.002
RN2	1.467	2.284	-1.644	-0.646	-0.553	1.558	-1.210	0.180	-0.471	-1.021	-0.288	0.116	0.061
RN3	0.701	2.066	-0.716	-0.999	-1.123	0.736	0.435	0.019	0.229	0.564	-0.202	0.128	0.355
RN4	0.997	1.114	-2.110	-0.734	-0.545	0.827	-0.369	0.202	0.698	0.135	0.819	0.058	-0.203

Only the six metabolites with the largest loading vectors in the PC1–PC2 plane are labelled in the PCA biplots for graphical clarity. Complete PCA scores and loadings for all included metabolites are provided in the present supplementary tables. Sample codes indicate cultivar, treatment and biological replicate: IB = 'Istarska bjelica'; R = 'Rosinjola'; B45 and I7 = bacterial isolates; N = negative control. The final numeral denotes the biological replicate.
