# Supplementary material for: Long-Term Metabolic Responses of Olive to Bacterial and Fungal Inoculation Differ Between Cultivars
Source: Biomolecules. 2026 Aug 21;16(8):1220. doi: 10.3390/biom16081220 (PMC13511578; doi:10.3390/biom16081220)
Supplement: Supplementary file 1 [file biomolecules-16-01220-s001.zip › Supplementary Table S5.pdf]

Supplementary Table S5. Principal component analysis (PCA) loadings of metabolites from the bacterial experiment.

Metabolite	PC1	PC2	PC3	PC4	PC5	PC6	PC7	PC8	PC9	PC10	PC11	PC12	PC13
Fructose	-0.170	-0.316	0.119	-0.126	-0.240	0.654	0.283	0.267	-0.182	0.055	0.322	-0.184	-0.175
Mannitol	-0.445	-0.309	-0.101	0.028	-0.019	0.065	-0.174	-0.040	-0.183	-0.027	-0.381	0.608	-0.330
Glucose	0.481	-0.054	0.063	-0.139	-0.065	0.372	-0.102	-0.066	0.069	0.372	-0.038	0.510	0.425
Sucrose	-0.370	-0.358	-0.154	-0.213	-0.065	0.045	-0.327	-0.198	-0.026	-0.009	-0.153	-0.360	0.600
Quinic acid	-0.225	0.229	0.222	-0.380	-0.363	-0.019	0.487	-0.096	0.407	-0.182	-0.314	0.112	0.119
Malic acid	-0.167	0.277	-0.014	-0.378	0.391	0.124	-0.288	0.652	0.221	0.100	-0.125	-0.025	-0.020
Ascorbic acid	-0.164	0.019	-0.008	0.563	0.280	0.446	-0.006	-0.169	0.577	-0.108	-0.053	-0.034	0.035
Aspartic acid	0.255	0.323	-0.324	-0.148	-0.215	0.379	-0.307	-0.130	-0.145	-0.594	-0.112	-0.045	-0.119
Glutamine	-0.190	0.133	0.594	-0.241	0.094	0.025	-0.394	-0.386	0.042	-0.077	0.431	0.109	-0.129
Alanine	0.009	0.020	-0.182	-0.340	0.669	0.157	0.410	-0.393	-0.228	0.005	-0.055	-0.023	-0.033
Glutamic acid	-0.224	0.412	-0.362	-0.035	-0.259	0.097	-0.091	-0.282	0.048	0.636	0.041	-0.138	-0.237
Lysine	-0.086	0.363	0.468	0.298	0.027	0.183	0.015	0.031	-0.505	0.110	-0.444	-0.163	0.165
Tryptophan	-0.375	0.348	-0.240	0.171	0.005	-0.031	0.162	0.139	-0.219	-0.166	0.457	0.362	0.434

Only the six metabolites with the largest loading vectors in the PC1–PC2 plane are labelled in the PCA biplots for graphical clarity. Complete PCA scores and loadings for all included metabolites are provided in the present supplementary tables.
