# Supplementary material for: Long-Term Metabolic Responses of Olive to Bacterial and Fungal Inoculation Differ Between Cultivars
Source: Biomolecules. 2026 Aug 21;16(8):1220. doi: 10.3390/biom16081220 (PMC13511578; doi:10.3390/biom16081220)
Supplement: Supplementary file 1 [file biomolecules-16-01220-s001.zip › Supplementary Table S6.pdf]

Supplementary Table S6. Principal component analysis (PCA) scores for individual samples from the fungal experiment.

Sample	PC1	PC2	PC3	PC4	PC5	PC6	PC7	PC8	PC9	PC10	PC11	PC12
IBNP_1	-1.757	-0.596	0.159	-2.120	1.639	0.841	-0.974	0.942	-0.598	0.637	1.437	0.266
IBNP_2	-0.885	-2.254	-1.238	0.313	-0.451	-0.135	-0.215	-0.234	-1.031	-0.863	-1.039	-1.228
IBNP_3	-1.479	-0.173	0.861	-1.469	0.630	0.185	-0.770	0.105	1.045	-0.358	-1.180	-0.715
IBNP_4	1.302	-1.918	-2.538	0.511	-1.581	-0.689	-0.200	-0.338	-0.707	-0.038	-0.079	0.279
IBN_1	1.142	-1.882	-0.696	-1.196	-0.175	0.338	-0.350	-0.499	0.847	-2.285	0.178	-0.241
IBN_2	-0.097	-3.671	-0.558	-2.528	0.981	-0.235	-0.728	0.186	-1.038	0.207	1.559	0.608
IBN_3	-1.763	-1.326	-0.793	-2.283	0.762	-0.143	0.191	0.331	-0.137	0.241	-1.911	-0.148
IBN_4	2.291	1.307	0.818	2.111	4.901	-1.567	0.779	0.553	-0.276	-1.504	0.332	0.235
IBDOI_1	0.797	0.189	0.458	0.857	1.805	-0.850	-0.935	0.065	-0.006	2.499	-0.594	-0.355
IBDOI_2	3.927	-3.126	2.070	1.666	-0.567	-1.047	-0.118	-0.437	0.831	0.645	0.642	-1.724
IBDOI_3	0.830	0.259	-2.808	0.599	0.696	0.151	0.791	-0.656	0.833	0.531	-0.534	0.393
IBDOI_4	2.287	-0.541	-3.512	0.840	-0.986	-0.017	0.903	-0.208	1.510	0.409	0.373	0.871
BNP_1	2.054	4.047	0.521	-2.824	-1.720	-4.193	-0.207	-0.045	-0.303	-0.078	-0.210	0.497
BNP_2	-0.240	1.970	-1.470	1.465	0.987	1.296	-0.661	-0.578	-2.055	0.366	-0.849	-0.009
BNP_3	-2.193	1.625	1.000	-1.170	0.010	0.459	-0.162	1.095	1.588	-0.133	0.136	-0.819
BNP_4	2.776	4.733	-1.778	-0.649	-0.598	2.514	-1.221	-0.093	-0.134	-0.195	1.047	-1.158
BN_1	-3.182	0.332	1.644	0.803	-0.964	-0.055	-1.305	-1.788	0.185	0.561	0.458	-0.089
BN_2	-2.612	-0.014	-0.445	2.935	-1.818	-0.634	-1.174	4.109	0.199	-0.275	0.114	0.364
BN_3	-2.116	0.184	0.753	1.606	-0.643	-0.719	-0.502	-2.000	0.618	0.023	0.572	0.592
BN_4	-3.205	0.748	1.313	1.430	-0.477	0.209	-0.598	-1.578	-0.549	-1.169	-0.074	0.719
BDOI_1	4.166	-0.735	3.133	-0.058	-2.047	1.312	0.640	0.658	-2.004	-0.039	-0.442	0.606
BDOI_2	2.678	0.231	2.521	-0.294	-0.009	2.241	0.651	0.264	1.445	0.046	-0.762	1.036
BDOI_3	-1.653	-0.347	0.473	-0.512	0.348	0.864	1.548	-0.006	0.524	0.531	0.067	1.137
BDOI_4	-3.067	0.955	0.111	-0.033	-0.723	-0.127	4.619	0.150	-0.786	0.241	0.760	-1.118

Supplementary Table S6. Principal component analysis (PCA) scores for individual samples from the fungal experiment. (continued)

Sample	PC13	PC14	PC15	PC16	PC17	PC18	PC19	PC20	PC21	PC22	PC23	PC24
IBNP_1	-0.523	0.803	0.458	-0.291	0.908	0.345	-0.010	-0.028	-0.199	0.068	0.007	0.000
IBNP_2	0.325	0.662	-1.192	0.623	0.696	0.006	-0.225	-0.051	-0.031	-0.066	-0.030	0.000
IBNP_3	-0.260	-0.331	0.169	-0.599	0.261	-0.973	-0.014	-0.129	-0.029	-0.003	0.087	0.000
IBNP_4	-0.962	-0.920	-0.214	-1.062	-0.101	0.111	0.260	-0.267	-0.239	-0.033	-0.051	0.000
IBN_1	1.137	-0.081	0.171	-0.728	-0.049	0.312	0.001	0.426	0.139	0.079	-0.013	0.000
IBN_2	0.620	-0.174	-0.255	0.425	-0.610	-0.357	0.106	-0.204	0.237	-0.060	0.010	0.000
IBN_3	-0.385	-0.666	0.569	0.870	-0.463	0.554	0.137	0.056	-0.152	0.063	0.023	0.000
IBN_4	-0.568	-0.531	-0.145	0.151	0.060	0.076	0.023	0.008	-0.049	-0.052	0.012	0.000
IBDOI_1	0.520	-0.533	-0.798	-0.407	0.066	0.038	-0.086	0.129	0.208	0.132	-0.034	0.000
IBDOI_2	0.003	0.633	0.780	0.159	-0.377	0.015	-0.218	-0.089	-0.167	0.008	-0.001	0.000
IBDOI_3	0.315	0.112	1.308	0.324	0.555	-0.327	0.173	-0.041	0.213	-0.057	-0.080	0.000
IBDOI_4	-1.114	0.446	-0.496	0.221	0.033	0.136	-0.281	0.069	0.125	0.037	0.099	0.000
BNP_1	0.474	0.471	0.033	-0.020	0.054	0.041	-0.137	-0.043	-0.026	-0.020	-0.002	0.000
BNP_2	0.453	1.305	0.302	-0.582	-0.631	0.063	0.104	0.085	-0.005	-0.062	0.044	0.000
BNP_3	-1.627	0.728	-0.444	-0.120	-0.479	0.094	0.312	0.080	0.180	-0.065	-0.069	0.000
BNP_4	0.275	-0.848	-0.122	0.528	-0.103	-0.135	-0.100	-0.041	-0.121	0.032	-0.009	0.000
BN_1	-0.074	-0.947	0.271	-0.056	0.275	0.511	-0.238	0.138	0.129	-0.178	0.040	0.000
BN_2	0.755	-0.202	0.266	0.139	-0.036	-0.032	-0.002	0.005	-0.001	0.004	0.010	0.000
BN_3	0.375	0.239	-0.404	0.505	0.058	-0.269	0.866	0.175	-0.215	0.061	0.012	0.000
BN_4	-0.645	0.153	0.244	0.136	-0.217	-0.061	-0.491	-0.364	0.142	0.156	-0.036	0.000
BDOI_1	-1.029	-0.224	0.151	0.196	0.288	-0.243	0.168	0.300	0.140	0.014	0.001	0.000
BDOI_2	1.140	0.187	-0.419	-0.111	0.092	0.433	0.165	-0.446	-0.026	-0.040	0.006	0.000
BDOI_3	0.301	-0.018	-0.246	-0.042	-0.328	-0.409	-0.645	0.336	-0.333	-0.054	-0.054	0.000
BDOI_4	0.496	-0.263	0.014	-0.259	0.046	0.072	0.131	-0.104	0.080	0.037	0.027	0.000

Only the six metabolites with the largest loading vectors in the PC1–PC2 plane are labelled in the PCA biplots for graphical clarity. Complete PCA scores and loadings for all included metabolites are provided in the present supplementary tables. Sample codes indicate cultivar, treatment and biological replicate: IB = ‘Istarska bjelica’; B = ‘Buža’; NP = *Neofusicoccum parvum*; DOI = *Dothiorella iberica*; N = negative control. The final numeral denotes the biological replicate.
