# Supplementary material for: Long-Term Metabolic Responses of Olive to Bacterial and Fungal Inoculation Differ Between Cultivars
Source: Biomolecules. 2026 Aug 21;16(8):1220. doi: 10.3390/biom16081220 (PMC13511578; doi:10.3390/biom16081220)
Supplement: Supplementary file 1 [file biomolecules-16-01220-s001.zip › Supplementary Table S7.pdf]

Supplementary Table S7. Principal component analysis (PCA) loadings of metabolites from the fungal experiment.

Metabolite	PC1	PC2	PC3	PC4	PC5	PC6	PC7	PC8	PC9	PC10	PC11	PC12
Sucrose	-0.061	-0.301	-0.193	-0.328	0.033	0.102	0.184	0.040	-0.296	-0.309	0.118	0.254
Glucose	-0.214	0.033	-0.010	-0.012	-0.069	0.073	0.596	0.047	-0.183	0.197	0.330	-0.200
Fructose	-0.234	-0.004	-0.013	0.456	-0.024	-0.025	-0.058	-0.163	-0.232	0.007	0.134	0.142
Quinic acid	-0.135	-0.261	0.019	0.042	-0.205	-0.075	-0.504	0.118	-0.160	-0.141	0.333	0.063
Malic acid	0.167	0.102	-0.224	0.219	0.251	0.420	-0.088	0.038	-0.199	0.192	-0.012	0.209
Citric acid	0.175	0.318	-0.291	-0.020	-0.170	0.145	-0.006	0.107	-0.095	0.086	0.218	-0.099
Succinic acid	0.103	0.394	-0.175	-0.035	-0.257	0.024	-0.025	-0.026	0.114	-0.051	0.266	0.165
Serine	0.289	-0.263	0.022	0.088	0.006	0.026	0.024	-0.282	0.187	0.314	0.204	0.014
Aspartic acid	0.185	-0.253	-0.224	0.298	0.037	-0.213	-0.105	-0.191	-0.111	-0.124	0.101	-0.050
Glutamine	-0.039	0.321	-0.125	0.138	0.286	-0.191	0.011	-0.037	-0.372	0.072	-0.180	-0.299
Alanine	0.095	0.166	0.077	-0.345	-0.274	-0.409	0.003	-0.183	-0.024	0.063	0.003	0.088
Glutamic acid	0.337	-0.067	-0.222	-0.146	0.160	-0.101	0.088	0.023	-0.150	-0.069	-0.034	-0.299
Lysine	0.088	0.203	-0.134	-0.042	0.051	0.486	-0.152	-0.227	0.168	-0.232	0.026	0.056
Proline	0.301	-0.025	0.284	-0.007	-0.007	0.133	0.237	0.151	-0.082	-0.047	-0.205	0.420
Histidine	0.179	-0.045	0.467	0.170	-0.020	0.203	0.090	0.084	0.111	-0.053	0.114	-0.150
Arginine	0.332	-0.103	0.226	0.059	-0.202	0.087	0.060	0.001	-0.183	0.115	0.012	-0.193
Valine	0.347	-0.160	-0.101	0.092	-0.045	-0.059	0.065	0.110	0.036	-0.130	0.381	-0.172
Isoleucine	0.224	0.053	-0.159	0.283	0.014	-0.359	0.038	0.094	0.200	0.306	-0.115	0.353
Leucine	-0.042	-0.174	-0.428	0.014	-0.128	-0.024	0.365	-0.055	0.228	-0.119	-0.068	0.183
Phenylalanine	0.293	-0.100	-0.127	-0.290	0.180	-0.003	-0.205	0.034	0.050	-0.057	-0.220	-0.153
Tryptophan	0.208	0.329	0.115	-0.094	-0.255	-0.077	-0.092	-0.010	-0.271	-0.071	0.097	0.145
Riboflavin	0.141	0.118	0.203	0.095	0.396	-0.232	0.150	-0.066	-0.208	-0.409	0.151	0.262
Biotin	-0.051	0.220	0.074	-0.033	0.411	-0.149	0.007	0.142	0.461	-0.148	0.383	-0.063

Only the six metabolites with the largest loading vectors in the PC1–PC2 plane are labelled in the PCA biplots for graphical clarity. Complete PCA scores and loadings for all included metabolites are provided in the present supplementary tables.

Supplementary Table S7. Principal component analysis (PCA) loadings of metabolites from the fungal experiment. (continued)

Metabolite	PC13	PC14	PC15	PC16	PC17	PC18	PC19	PC20	PC21	PC22	PC23	PC24
Sucrose	0.132	0.128	-0.128	0.111	-0.152	0.079	0.101	0.036	0.284	0.419	0.141	-0.102
Glucose	0.093	0.110	0.140	-0.404	0.118	0.176	0.141	-0.229	-0.054	-0.074	-0.144	0.121
Fructose	-0.016	0.499	-0.164	0.055	-0.333	0.041	-0.379	0.112	-0.108	-0.148	-0.075	0.077
Quinic acid	-0.054	0.052	0.073	-0.105	0.385	0.373	0.121	0.086	0.169	-0.166	-0.095	-0.050
Malic acid	-0.234	0.122	0.095	0.001	0.037	0.131	0.089	-0.299	-0.184	0.222	0.197	-0.239
Citric acid	-0.354	0.035	0.101	0.136	-0.089	-0.223	0.076	0.025	0.509	-0.345	0.089	-0.013
Succinic acid	-0.124	-0.114	-0.359	-0.216	0.305	-0.046	-0.303	0.120	-0.125	0.408	-0.086	0.154
Serine	0.234	-0.123	-0.102	0.061	-0.118	0.056	-0.281	-0.240	0.467	0.095	-0.249	-0.016
Aspartic acid	-0.066	-0.333	0.350	-0.112	0.039	-0.026	-0.108	-0.256	-0.142	0.082	0.244	0.236
Glutamine	0.279	0.001	-0.042	0.346	0.336	0.190	-0.033	0.093	0.215	0.144	0.018	0.217
Alanine	0.048	0.370	0.296	-0.021	-0.016	0.004	-0.190	-0.147	0.010	0.043	0.436	0.011
Glutamic acid	0.002	-0.095	-0.198	-0.305	-0.107	0.281	-0.302	0.281	-0.109	-0.248	0.124	-0.397
Lysine	0.542	0.096	0.321	-0.197	-0.018	0.017	0.001	0.212	0.010	-0.137	0.042	0.144
Proline	-0.132	-0.104	-0.091	0.024	0.043	0.373	-0.104	0.032	0.082	-0.272	0.135	0.464
Histidine	0.107	0.218	-0.041	0.138	0.425	-0.076	-0.162	-0.101	0.029	0.014	0.256	-0.350
Arginine	-0.195	0.087	0.406	0.072	-0.120	0.027	0.042	0.491	-0.077	0.372	-0.259	0.053
Valine	0.181	0.135	-0.308	0.250	-0.062	-0.195	0.321	0.032	-0.284	-0.102	0.176	0.275
Isoleucine	0.203	0.187	-0.026	-0.220	0.059	0.076	0.402	0.201	0.098	0.043	0.001	-0.170
Leucine	-0.055	0.055	0.234	0.426	0.326	0.046	-0.185	0.087	-0.167	-0.173	-0.217	-0.154
Phenylalanine	-0.134	0.491	-0.059	-0.112	0.135	-0.008	-0.009	-0.313	-0.056	-0.013	-0.403	0.217
Tryptophan	0.250	-0.195	0.021	0.257	-0.196	0.196	0.104	-0.306	-0.259	-0.107	-0.330	-0.263
Riboflavin	-0.068	-0.011	0.129	-0.180	0.112	-0.403	-0.015	0.023	0.128	-0.078	-0.243	-0.114
Biotin	-0.169	0.052	0.172	0.170	-0.227	0.426	-0.014	0.003	0.011	0.130	0.006	0.027

Only the six metabolites with the largest loading vectors in the PC1–PC2 plane are labelled in the PCA biplots for graphical clarity. Complete PCA scores and loadings for all included metabolites are provided in the present supplementary tables.
